# Transport of Small Aliphatic Amines by Polyspecific Solute Carriers: Deciphering Structure–Function Relationships

**DOI:** 10.1021/acsptsci.5c00340

**Published:** 2025-07-16

**Authors:** Wouroud Ismail Al-Khalil, Jürgen Brockmöller, Muhammad Rafehi

**Affiliations:** Institute of Clinical Pharmacology, University Medical Center Göttingen, D-37075 Göttingen, Germany

**Keywords:** organic cation transporters, solute carriers, aliphatic amines, structure−function relationship analysis, ethanolamine, polyamines

## Abstract

Membrane proteins of the solute carrier (SLC) 22A and 47A families are polyspecific transporters critical for handling diverse endogenous and exogenous compounds, including many clinically relevant drugs. However, their substrate specificity remains poorly understood. To address this, we conducted a structure–function relationship analysis focusing on small aliphatic amines (alkylamines and alkanolamines) and enantioselectivity of their transport. Using HEK293 cells stably overexpressing organic cation transporters (OCT) 1, 2, or 3 (*SLC22A1*, *-2*, or *-3*) or multidrug and toxin extrusion transporters (MATE) 1 or 2-K (*SLC47A1* and *-2*), substrate transport was quantified through liquid chromatography—tandem mass spectrometry, with 6-aminoquinolyl-*N*-hydroxysuccinimidyl carbamate derivatization as needed. While most tested compounds exhibited physicochemical properties consistent with typical OCT and MATE substrates, compounds with a molecular weight of less than 145 Da were mostly not transported by OCT1, OCT3, or the MATEs. However, in great contrast, OCT2 demonstrated robust transport of small aliphatic amines and alkanolamines (molecular weight of 60–145 Da), with modest stereoselectivity favoring (*S*)-enantiomers. Structural complexity, such as carbon chain length and amino group positioning, strongly influenced transport activity and kinetics, while compounds with more than two positive charges at different positions were not transported. Additionally, we identified a novel role for OCT2 as an efficient efflux transporter for ethanolamine. These findings reveal critical molecular determinants underlying SLC-mediated transport, enhancing our understanding of OCT and MATE substrate preferences and mechanisms. This knowledge provides a foundation for better predicting transporter interactions and optimizing drug design.

## Graphical abstract

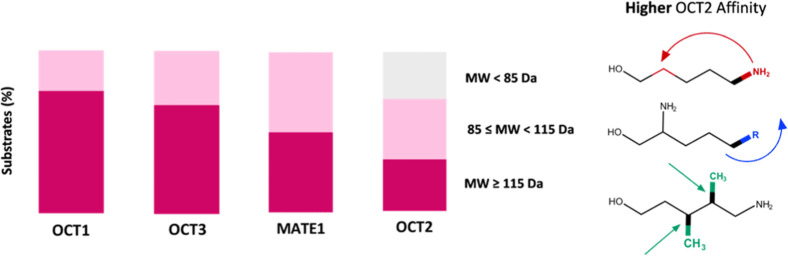

1

Organic cations comprise approximately one-third of all biologically significant small molecules and around 40% of all commonly utilized drugs.^1,2^ Due to their hydrophilicity and positive charge at physiological pH, passive diffusion across biological membranes is limited, and their passage is facilitated by membrane transporters. While organic cations may pass membranes by a process called nonionic diffusion, this is particularly slow for substances with a p*K*_abasic_ above 8.

Transporters for organic cations are polyspecific uniporters or antiporters belonging to the solute carriers (SLCs) transporters. Most distinct protein members are the organic cation transporters 1–3 (OCTs 1–3, *SLC22A1*, 2, and 3), as well as the multidrug and toxin extrusion 1 (MATE1, *SLC47A1*) and MATE2 kidney-specific (MATE2-K, *SLC47A2*). They facilitate the cellular uptake or extrusion of numerous structurally diverse cationic compounds. Widely expressed across various epithelial tissues throughout the human body, these transporters are directly involved in drug disposition and pharmacokinetics, and they may contribute to modulating the distribution of cationic endogenous compounds.^1,3^

Due to their presumed clinical relevance, intensive efforts have been made over the last decades to identify the substrate spectra of OCTs and MATEs. Additionally, considerable progress has been achieved in solving the molecular structures of some of these OCTs,^4–6^ which may assist in the prediction of which substances are substrates of a specific OCT. However, the currently available cryogenic electron microscopy structures do not allow to reliably differentiate between substrates and inhibitors. Consequently, the U.S. Food and Drug Administration and the European Medicines Agency guidelines still recommend using in vitro models as tools for identifying substrates during drug development.^7^ Therefore, experimental validation remains crucial.

Polyspecificity is a particularly prominent feature of OCTs and MATEs, and the substrate spectra broadly overlap among individual transporters within and between these SLC families.^1,8^ However, polyspecificity does not imply a lack of specificity. For instance, notable variations in OCT1 interaction with different opioids with subtle changes in chemical structure have been observed.^9^ Moreover, varying substrate specificities among the closely related OCTs have been reported even within the same drug class, such as triptans.^10^ Altogether, this suggests that minor structural substitutions and certain functional groups can substantially influence OCT specificity.

Typical OCT substrates generally exhibit similar physicochemical properties in terms of molecular weight (*M*_W_ < 600 Da), lipophilicity (Log *D*_pH7.4_ < 1.5), and positive charge at physiological pH.^11,12^ However, within these limits, earlier studies could not identify specific pharmacophores capable of predicting which substances act as substrates for particular transporters. As a result, substrate specificity remains poorly understood, and structural determinants for specific transporter–substrate interactions are lacking. Enhanced knowledge of the structure–function relationships of these transporters and the elucidation of specific molecular features will aid in predicting drug transport for drug discovery and pharmacokinetic studies.

Comprehensive studies to identify novel OCT substrates were mostly conducted with drugs.^11,12^ In this study, we focus on small and hydrophilic organic cations with physicochemical properties that are in accordance with those of typical OCT substrates. Comparing the transport activity over a narrow chemical spectrum provides the opportunity to attribute the observed variations to substrate structure rather than general chemical features. Furthermore, racemic substances (chiral compounds) are of particular interest owing to the fact that their enantiomers share identical physicochemical properties but differ in three-dimensional structure. Recently, stereoselectivity in OCT-mediated cellular uptake was investigated, and some enantiomers were transported by OCTs with moderate to high stereoselectivity.^5,13–15^ Nevertheless, no specific molecular features could be identified to predict transport potential or stereoselectivity.^15^ Most substances tested so far had at least one cyclic structure, often more rigid aromatic structures. Accordingly, we hypothesized that the structurally more flexible structures might exhibit distinct transporter selectivity and stereoselectivity. Therefore, compounds were selected to comprehensively study all alkylamines and hydroxylated alkylamines (alkanolamines) from aliphatic hydrocarbons containing 2 to 7 carbon atoms (Figure 1).

**Figure 1:**
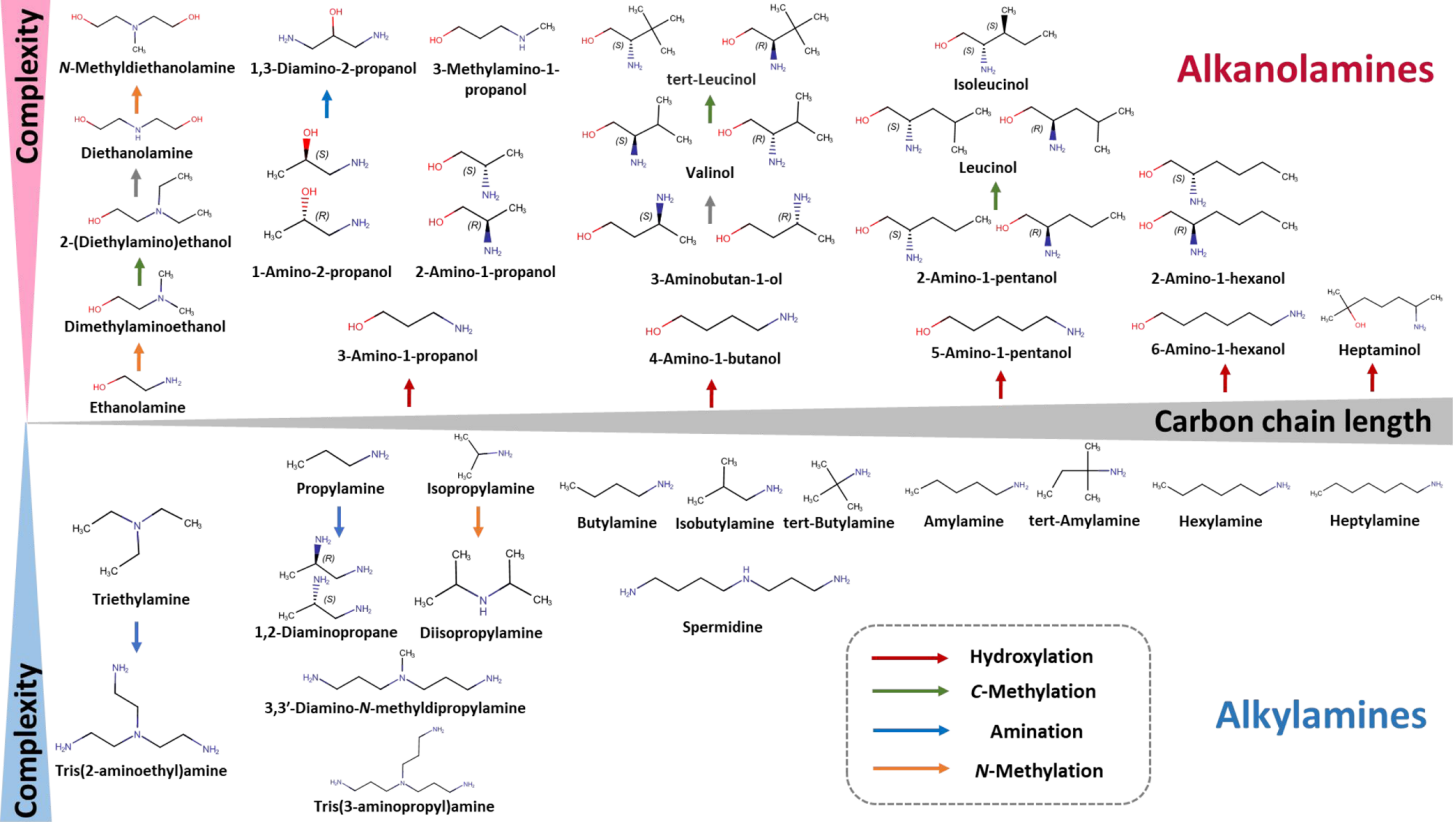
Chemical structures of investigated aliphatic amines, classified into two major categories, alkanolamines and alkylamines.

## 2 Results

In this study, we screened 46 small aliphatic amines for cellular uptake by transporters of the SLC22A- and SLC47A-families. The selection criteria included a positive charge at neutral pH, the presence of at least one primary, secondary, or tertiary amino group, and a carbon chain length of fewer than eight carbon atoms. A large subgroup was alkanolamines, as the hydroxyl group would increase the hydrophilicity of the molecules, thereby enhancing the necessity for transporter-mediated uptake (Figure 1). Initially, we identified which substances were transported by which of the five transporters and compared the kinetic parameters of shared and transporter-specific substrates. Next, we examined the extent of stereoselectivity in SLC-mediated transport of aliphatic amines. Lastly, we conducted a comprehensive analysis of how the chemical properties influence the transport potential via these SLC transporters.

### 2.1 Transport of Short-Chain Aliphatic Amines by OCTs 1–3 and MATEs

Although the test compounds all have typical physicochemical properties of known substrates, only a small number of these exhibited OCT1-, OCT3-, and MATE1-mediated uptake with an uptake ratio ≥2 (Figures 2 and 3, Table 1). Specifically, this had been the case for merely four (9%, OCT1), three (7%, OCT3), and four (9%, MATE1) substances. None of the studied aliphatic amines showed notable uptake in MATE2-K-overexpressing cells, despite MATE2-K activity having been confirmed with approximately 100 other substrates in our laboratory^16^ [Redeker et al., unpublished data]. In stark contrast, OCT2 demonstrated very broad transport activity, as 37 (80%) of the aliphatic amines were transported by OCT2 with an uptake ratio ≥2.

**Figure 2:**
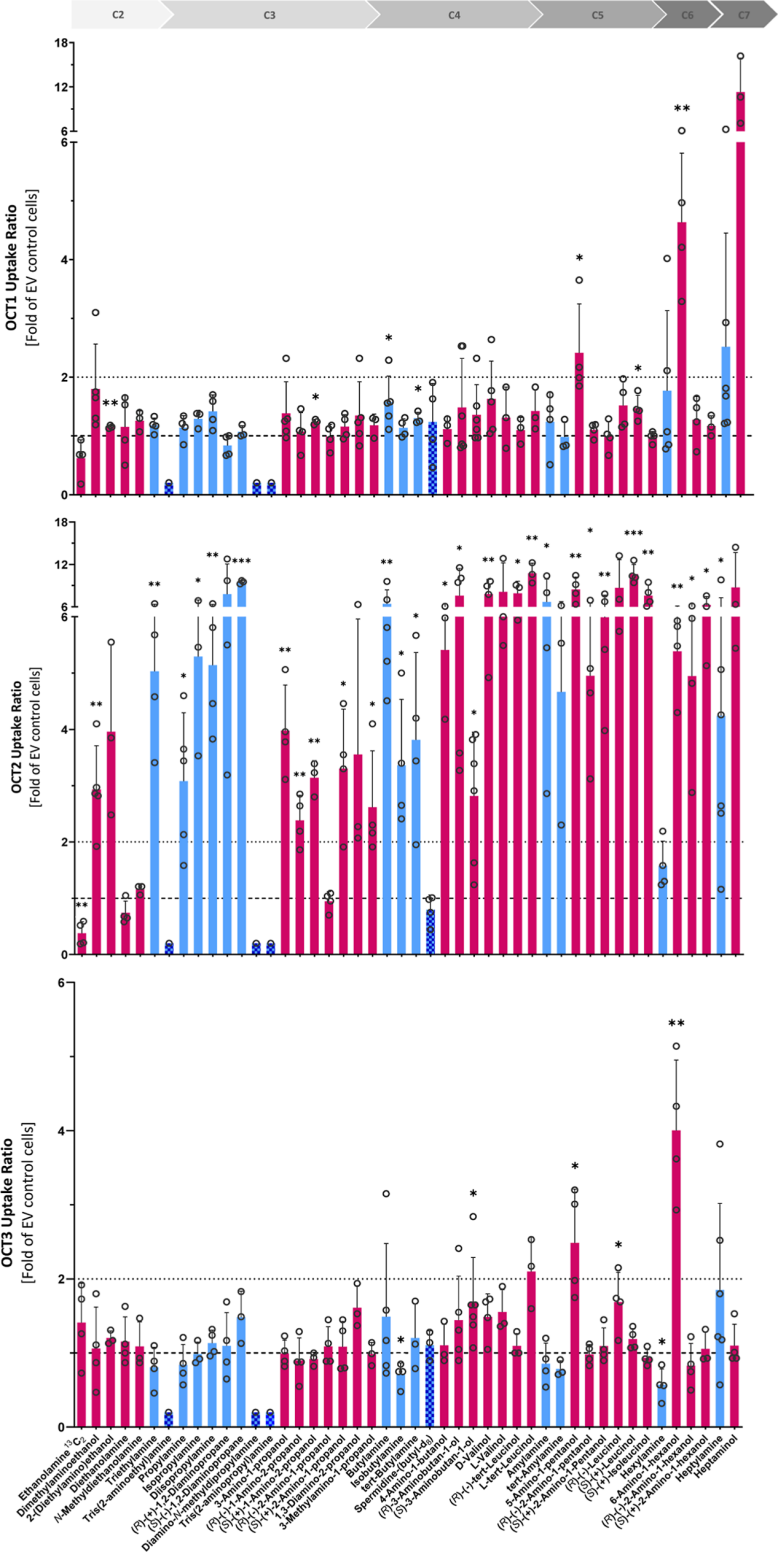
Transport of aliphatic amines by OCTs 1–3, shown as uptake ratios into transporter-overexpressing cells over EV control cells. Test substances are ordered by carbon chain length. The lower dashed lines represent an uptake ratio of 1 (no transporter-specific uptake), the upper dashed lines of 2 (threshold for notable transport). Alkylamines, alkanolamines, and amines with ≥3 amino groups are shown in blue, dark pink, and checker pattern bars, respectively. Individual data points are shown and bar charts represent the means ± SD of ≥3 independent experiments. Asterisks denote statistical significance compared to EV control cells (one-sample *t*-test, *p < 0.05, **p < 0.01, and ***p < 0.001).

**Figure 3:**
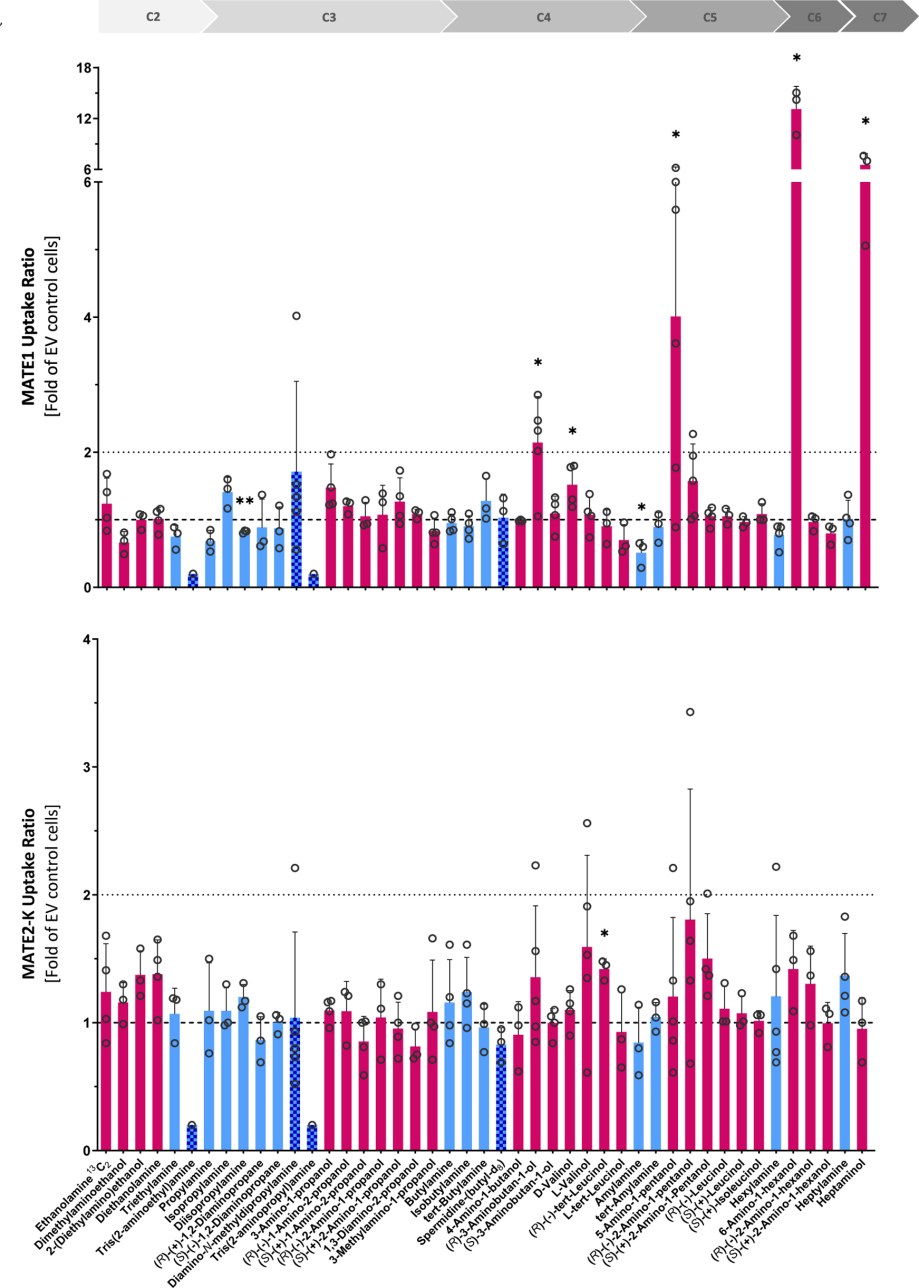
Transport of aliphatic amines by MATEs 1 and 2-K, shown as uptake ratios into transporter-overexpressing cells over EV control cells. Test substances are ordered by carbon chain length. The lower dashed lines represent an uptake ratio of 1 (no transporter-specific uptake), the upper dashed lines of 2 (threshold for notable transport). Alkylamines, alkanolamines, and amines with ≥3 amino groups are shown in blue, dark pink, and checker pattern bars, respectively. Individual data points are shown and bar charts represent the means ± SD of ≥3 independent experiments. Asterisks denote statistical significance compared to EV control cells (one-sample *t*-test, *p < 0.05, **p < 0.01, and ***p < 0.001).

**Table 1:** Transport Ratios for Transporter-Overexpressing Cells

	uptake ratios: mean (±SD, N)				
compound	OCT1	OCT2	OCT3	MATE1	MATE2K
ethanolamine^13^C_2_	0.6 (0.3, 4)	0.4 (0.2, 4)^b^	1.4 (0.5, 4)	1.2 (0.4, 4)	1.2 (0.4, 4)
dimethylaminoethanol	1.8 (0.8, 5)	2.9 (0.8, 5)^b^	1.1 (0.6, 4)	0.66 (0.16, 3)	1.2 (0.2,3)
2-(diethylamino)ethanol	1.2 (0.03, 3)^b^	4.0 (1.5, 3)	1.2 (0.1, 3)	1 (0.13, 3)	1.4 (0.2, 3)
diethanolamine	1.2 (0.5, 4)	0.7 (0.2, 4)	1.2 (0.3, 4)	1.0 (0.2, 4)	1.4 (0.3, 4)
*N*-methyldiethanolamine§	1.3 (0.2, 3)	1.2 (0.1, 3)	1.1 (0.3, 3)	not tested	
triethylamine	1.2 (0.1, 4)	5.0 (1.3, 4)^b^	0.8 (0.3, 4)	0.8 (0.2, 3)	1.1 (0.2, 3)
tris(2-aminoethyl)amine	-^d^	-^d^	-^d^	-^d^	-^d^
propylamine	1.1 (0.2, 4)	3.1 (1.2, 5)^a^	0.8 (0.3, 4)	0.7 (0.2, 3)	1.1 (0.4, 3)
isopropylamine	1.3 (0.2, 3)	5.3 (1.7, 3)^a^	1.0 (0.2, 3)	1.4 (0.2, 3)	1.1 (0.2, 0.3)
diisopropylamine	1.4 (0.3, 4)	5.1 (1.2, 4)^b^	1.1 (0.2, 4)	0.8 (0.02, 3)^b^	1.2 (0.1, 3)
(*R*)-(+)-1,2-diaminopropane	0.8 (0.2, 4)	7.8 (4.3, 4)	1.1 (0.5, 4)	0.9 (0.4, 3)	0.9 (0.2, 3)
(S)-(−)-1,2-diaminopropane	1.1 (0.1, 3)	9.5 (0.2, 3)^c^	1.5 (0.4, 3)	0.9 (0.3, 3)	1.0 (0.1, 3)
3,3′-diamino-*N*-methyldipropylamin	-^d^	-^d^	-^d^	1.7 (1.3, 5)	1.0 (0.7, 5)
tris(2-aminopropyl)amine	-^d^	-^d^	-^d^	-^d^	-^d^
3-amino-1-propanol	1.4 (0.5, 5)	4.0 (0.8, 4)^b^	1.0 (0.2, 4)	1.5 (0.4, 4)	1.1 (0.1, 4)
(*R*)-(−)-1-amino-2-propanol	1.1 (0.3, 4)	2.4 (0.5, 4)^b^	0.9 (0.3, 4)	1.2 (0.1, 3)	1.1 (0.2, 3)
(*S*)-(+)-1-amino-2-propanol	1.2 (0.1, 3)^a^	3.1 (0.3, 3)^b^	0.9 (0.1, 3)	1.1 (0.2, 3)	0.9 (0.2, 4)
(*R*)-(−)-2-amino-1-propanol	1.0 (0.2, 4)	1.0 (0.2, 4)	1.1 (0.3, 4)	1.1 (0.4, 3)	1.0 (0.3, 3)
(*S*)-(+)-2-Amino-1-propanol	1.2 (0.2, 4)	3.3 (1.7, 4)^a^	1.1 (0.3, 4)	1.3 (0.4, 4)	1.0 (0.2, 4)
1,3-diamino-2-propanol	1.4 (0.6, 5)	3.7 (2.4, 3)	1.6 (0.3, 3)	1.1 (0.1, 3)	0.8 (0.1, 3)
3-methylamino-1-propanol	1.2 (0.2, 3)	2.6 (1.0, 4)^a^	1.0 (0.2, 3)	0.8 (0.2, 4)	1.1 (0.4, 4)
butylamine	1.6 (0.4, 5)^a^	6.4 (2.0, 5)^b^	1.5 (1.0, 5)	1.0 (0.1, 4)	1.2 (0.3, 4)
isobutylamine	1.1 (0.2, 4)	3.4 (1.2, 4)^a^	0.7 (0.2, 4)^a^	0.9 (0.2, 4)	1.2 (0.3, 4)
tert-butylamine	1.3 (0.10, 3)^a^	3.8 (1.6, 4)^a^	1.3 (0.5, 3)	1.3 (0.3, 3)	1.0 (0.2, 3)
spermidine-(butyl-d_8_)§	1.2 (0.7, 4)	0.8 (0.3, 4)	1.1 (0.2, 4)	1.0 (0.4, 3)	0.8 (0.1, 3)
4-amino-1-butanol§	1.1 (0.3, 3)	5.4 (1.1, 3)^a^	1.1 (0.3, 3)	1.0 (0.01, 3)	0.9 (0.3, 3)
(*R*)-3-aminobutan-1-ol	1.5 (0.8, 6)	7.6 (3.9, 5)^a^	1.4 (0.6, 5)	2.1 (0.7, 5)^a^	1.4 (0.6, 5)
(*S*)-3-aminobutan-1-ol	1.4 (0.5, 6)	2.8 (1.1, 6)^a^	1.7 (0.6, 6)^a^	1.1 (0.3, 4)	1.0 (0.1, 4)
D-valinol§	1.6 (0.6, 5)	7.8 (2.1, 4)^b^	1.5 (0.3, 4)	1.5 (0.3, 4)^a^	1.1 (0.2, 4)
L-valinol§	1.3 (0.5,3)	8.1 (4.1, 3)	1.6 (0.3, 3)	1.1 (0.3, 4)	1.6 (0.8, 5)
(*R*)-(−)-tert-leucinol§	1.1 (0.2, 3)	7.9 (1.7, 3)^a^	1.1 (0.2, 3)	0.9 (0.2, 3)	1.4 (0.1, 3)^a^
L-tert-Leucinol§	1.4 (0.4, 3)	10.8 (1.4, 3)^b^	2.1 (0.5, 3)	0.7 (0.2, 3)	0.9 (0.3, 3)
amylamine	1.2 (0.5, 4)	6.7 (3.2, 4)^a^	0.9 (0.3, 4)	0.5 (0.2, 3)^a^	0.8 (0.3, 3)
*tert*-amylamine§	1.0 (0.3, 3)	4.7 (2.1, 3)	0.8 (0.1, 3)	0.9 (0.2, 3)	1.0 (0.1, 3)
5-amino-1-pentanol	2.4 (0.8, 4)^a^	8.5 (1.7, 4)^b^	2.5 (0.7, 4)^a^	4.0 (2.3, 6)^a^	1.2 (0.6, 5)
(*R*)-(−)-2-amino-1-pentanol§	1.1 (0.1, 4)	5.0 (1.6, 4)^a^	1.0 (0.1, 4)	1.6 (0.6, 5)	1.8 (1.0, 5)
(*S*)-(+)-2-amino-1-pentanol§	1.0 (0.3, 4)	6.0 (1.7, 4)^b^	1.1 (0.2, 4)	1.1 (0.1, 4)	1.5 (0.4, 4)
(*R*)-(−)-leucinol§	1.5 (0.4, 4)	8.7 (4.0, 3)	1.7 (0.4, 4)^a^	1.1 (0.1, 3)	1.1 (0.2, 3)
(*S*)-(+)-leucinol§	1.5 (0.2, 4)^a^	10.6 (1.4, 4)^c^	1.2 (0.1, 4)	1.0 (0.1, 3)	1.1 (0.1, 3)
(*S*)-(+)-isoleucinol§	1.0 (0.1, 4)	7.6 (1.5, 4)^b^	0.9 (0.1, 4)	1.1 (0.2, 3)	1.0 (0.1, 3)
hexylamine	1.8 (1.4, 5)	1.6 (0.4, 4)	0.6 (0.2, 4)^a^	0.8 (0.2, 4)	1.2 (0.6, 5)
6-amino-1-hexanol§	4.6 (1.2, 4)^b^	5.4 (0.8, 4)^b^	4.0 (1.0, 4)^b^	13.1 (2.7, 3)^a^	1.4 (0.3, 3)
(*R*)-(−)-2-amino-1-hexanol	1.3 (0.4, 4)	5.0 (1.5, 4)^a^	0.8 (0.3, 4)	1.0 (0.1, 3)	1.3 (0.3, 3)
(*S*)-(+)-2-amino-1-hexanol	1.2 (0.2, 3)	6.3 (1.2, 3)^a^	1.06 (0.23, 3)	0.8 (0.2, 3)	1.0 (0.2, 3)
heptylamine	2.5 (1.9, 6)	4.2 (3.1, 6)^a^	1.85 (1.17, 6)	1.0 (0.3, 4)	1.4 (0.3, 4)
heptaminol§	11.3 (4.6, 3)	8.7 (5.0, 3)	1.1 (0.29, 4)	6.6 (1.3, 3)^a^	1.0 (0.2, 3)

a Statistical significance compared to EV control cells using one sample *t*-test with *p* < 0.05,

b *p* < 0.01, and

c *p* < 0.001.

d - indicate no uptake in the respective SLC-overexpressing cells or EV-transfected control cells. Only substances indicated with § were tested for OCT-mediated uptake at a concentration of 2.5 μM. Otherwise, a concentration of 10 μM was applied. For MATE-mediated uptake, all investigated substances were tested at a concentration of 2.5 μM. N refers to the number of independent experiments performed for each substance for the respective transporter-overexpressing cells.

Generally, the uptake of nearly all aliphatic amines in OCT1-, OCT3-, MATE1-, and MATE2-K-overexpressing HEK293 cells was similar to that observed in empty vector (EV) control cells. For some substances, an uptake ratio below 2 was found to be statistically significant (one sample *t*-test, *p* < 0.05). For OCT1, uptake ratios of 1.2, 1.2, 1.3, 1.5, and 1.6 for 2-(diethylamino)­ethanol, (*S*)-(+)-1-amino-2-propanol, tert-butylamine, (*S*)-(+)-leucinol, and butylamine respectively, were statistically significant. This was also the case for the uptake ratio of 1.7 for (*R*)-(−)-leucinol and (*S*)-3-aminobutan-1-ol in OCT3, of 1.5 for d-valinol in MATE1, and of 1.4 for (*R*)-(−)-*tert*-leucinol in MATE2-K.

A few tested compounds showed a surprisingly lower uptake by transporter-overexpressing cells compared to EV control cells. This can best be explained by an efflux transport activity of some substrate–transporter pairs, in combination with passive non-ionic diffusion and/or influx transport via other unknown constitutively expressed transporters. For instance, ethanolamine exhibited 38% lower uptake in OCT1-transfected cells and 64% (one sample *t*-test, *p* = 0.009) less uptake in OCT2-transfected cells.

Interestingly, polyamines containing three or four amino groups, such as spermidine, tris­(2-aminoethyl)­amine, tris­(2-aminopropyl)­amine, and 3,3′-diamino-*N*-methyldipropylamin, did not show any substrate activity at any of the studied SLCs, including OCT2 (Fisher’s exact test, *p* = 0.0004). This was also the case for compounds with two hydroxyl groups (diethanolamine and *N*-methyldiethanolamine) for the OCTs and for diethanolamine for the MATEs (*N*-methyldiethanolamine had not been assessed at the MATEs).

### 2.2 Shared Substrates

The compounds 5-amino-1-pentanol and 6-amino-1-hexanol showed notable transport by OCTs 1–3 and MATE1-overexpressing cells and were thus shared substrates. Moreover, heptaminol was transported by OCT1, OCT2, and MATE1, illustrating a clear trend of higher transport activity across different SLC transporters with increasing length of the non-branched carbon chain. These three substrates were studied in greater detail in concentration-dependent experiments (Table 2).

**Table 2:** Kinetic Parameters for Transport of Identified Substrates

transporter	substrate	*N*	*K*_m_ [μM] (±SEM)	*v*_max_ [pmol/mg protein/min] (±SEM)	CL_int_ [μL/mg protein/min] (±SEM)
**OCT1**	heptaminol	4	241 (±68)	1480 (±174)	8 (±4)
	6-amino-1-hexanol	4	487 (±171)	1656 (±294)	3.4 (±1.8)
	5-amino-1-pentanol	3	132 (±121)	135 (±37)	1 (±1)
**OCT2**	heptaminol	4	1089 (±213)	9730 (±1210)	13 (±9)
	6-amino-1-hexanol	4	1176 (±273)	16,933 (±2577)	14 (±6)
	5-amino-1-pentanol	6	1260 (±452)	14,996 (±2181)	12 (±6)
	4-amino-1-butanol	5	1243 (±938)	3103 (±1458)	2.5 (±3)
	(*S*)-(+)-2-amino-1-hexanol	4	39 (±12)	786 (±72)^b^	20 (±8)
	(*R*)-(−)-2-Amino-1-hexanol	4	59 (±11)	1342 (±84)^b^	23 (±6)
	(*S*)-(+)-isoleucinol	4	116 (±59)	1303 (±287)	11 (±8)
	(*S*)-(+)-2-amino-1-pentanol	4	245 (±83)	2342 (±290)^a^	10 (±4)
	(*R*)-(−)-2-amino-1-pentanol	4	198 (±67)	1453 (±179)^a^	7 (±3)
	l-valinol	5	362(±156)	4970 (±967)	14 (±9)
	d-valinol	5	329 (±190)	2488 (±573)	8 (±6)
	diisopropylamine	4	68 (±32)	1388 (±157)	20 (±12)
	1,3-diamino-2-propanol	5	65 (±91)	110 (±56)	1.7 (±3)
	triethylamine	4	84 (±66)	1053 (±206)	13 (±12)
	2-(diethylamino)ethanol	4	57 (±28)	794 (±109)	14 (±9)
**OCT3**	6-amino-1-hexanol	4	1168 (±151)	4675 (±384)	4 (±1)
	5-amino-1-pentanol	5	5776 (±2458)	5271 (±1641)	0.9 (±0.7)
**MATE1**	heptaminol	3	154 (±18)	8061 (±283)	48 (±7)
	6-amino-1-hexanol	5	253 (±47)	14,115 (±1479)	56 (±16)
	5-amino-1-pentanol	3	1040 (±451)	10,761 (±2694)	10 (±7)

a Statistical significance of the differences between the two enantiomers using Student’s *t*-test with *p* < 0.05 and

b *p* < 0.01.

Concentration-dependent analyses confirmed high transport activity of heptaminol by OCT1 and OCT2, and highest by MATE1 (Figure 4). The latter is reflected in a Intrinsic clearance (CL_int_) of 48 μL/mg protein/min, which was 6.0- and 3.7-fold higher than that of OCT1 and OCT2 (one-way ANOVA, *p* = 0.009). Likewise, the maximum transport velocity (*v*_max_) of MATE1 was the highest (8458 pmol/mg protein/min), compared to 5847 and 1267 pmol/mg protein/min for OCT2 and OCT1, respectively (one-way ANOVA, *p* < 0.0001).

**Figure 4:**
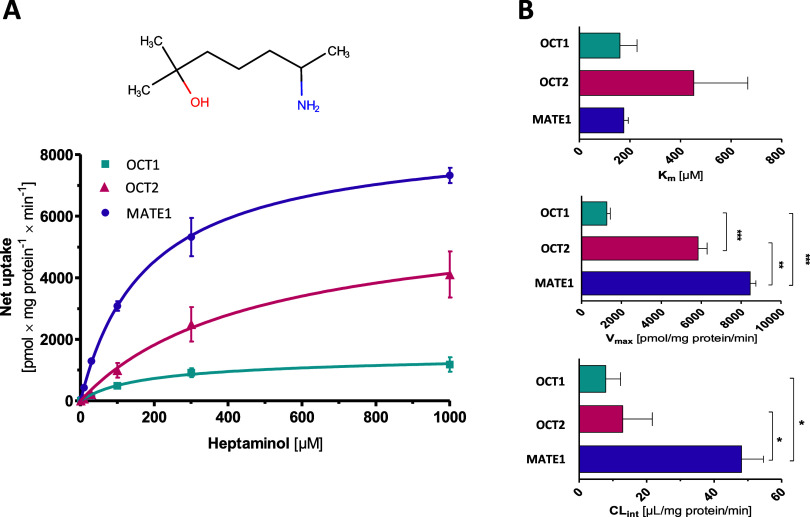
(A) Concentration-dependent net uptake of heptaminol by OCT1, OCT2, and MATE1. Data is shown as means ± SEM of ≥3 independent experiments. (B) Transport kinetic parameters of heptaminol by OCT1, OCT2, and MATE1. Differences were tested for statistical significance using one-way ANOVA test with Tukey’s post-hoc test, and indicated as *p < 0.05, **p < 0.01, and ***p < 0.001 refer to the results of Tukey’s post-hoc test.

The two shared substrates of OCTs 1–3 and MATE1, 5-amino-1-pentanol and 6-amino-1-hexanol, showed more than a 2-fold increase in uptake compared to EV control cells. However, testing concentration dependent uptake (Figure 5) revealed that these two substances were, in fact, poor OCT1 and OCT3 substrates (Table 2). In contrast, both substances were good MATE1 substrates, with a remarkably high intrinsic clearance (56 μL/mg protein/min) of 6-amino-1-hexanol.

**Figure 5:**
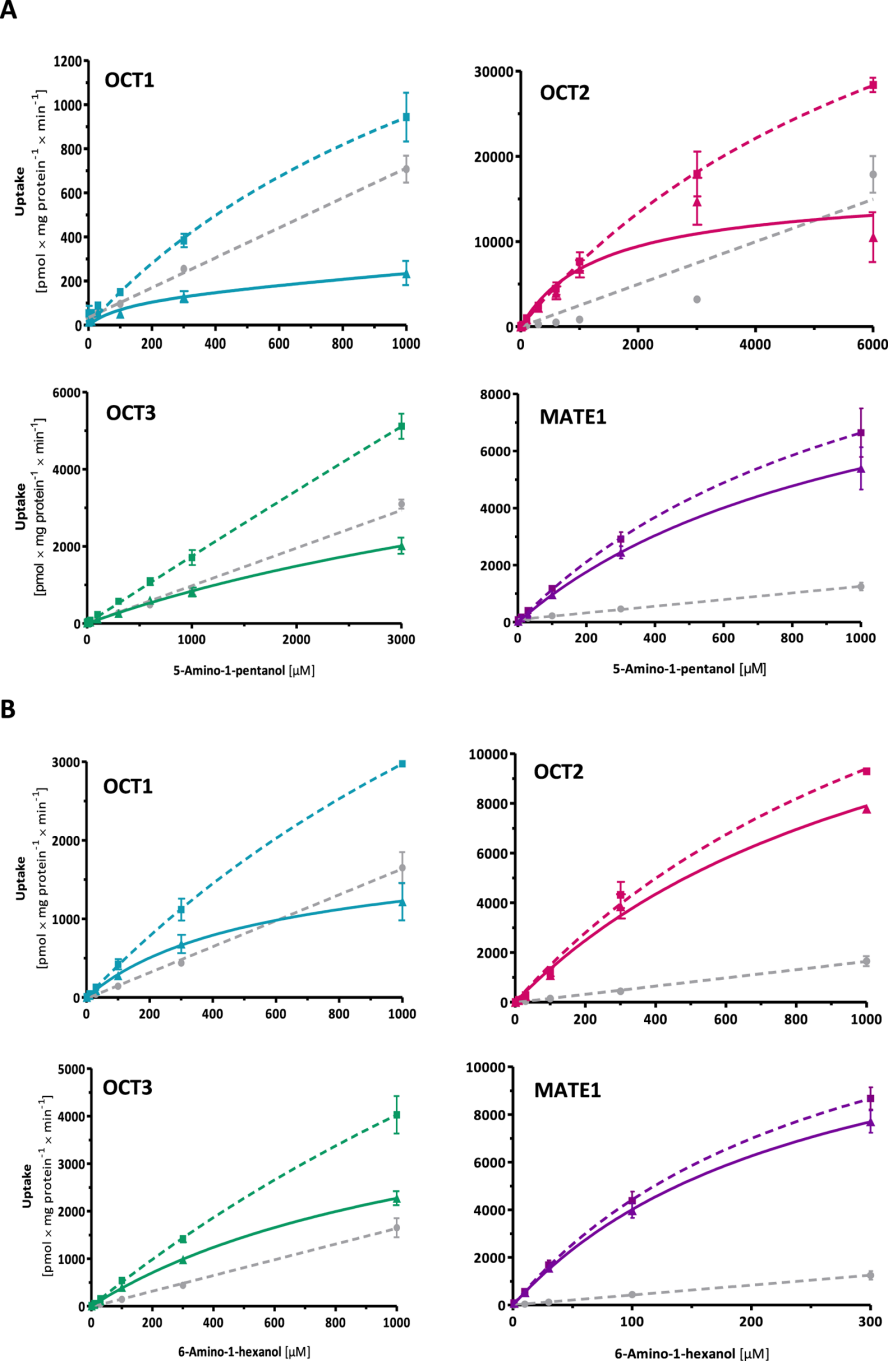
Concentration-dependent uptake of (A) 5-amino-1-pentanol and (B) 6-amino-1-hexanol by OCTs 1–3 and MATE1. Curves represent the total uptake by transporter-overexpressing cells (colored dotted curves), EV control cells (gray dotted curves), and net uptake by transporter-overexpressing cells (colored solid curves). Data is shown as means ± SEM of ≥3 independent experiments. The unexpected concentration uptake curve of 5-amino-pentanol at the high 6 mM concentration may be best explained by unspecific disturbance of membrane properties at such high concentration.

Notably, OCT2 mediated the uptake of 5-amino-1-pentanol and 6-amino-1-hexanol with comparatively low affinity but high capacity transport. Although the total uptake of 5-amino-pentanol by OCT2 was significantly higher than that of EV control cells, saturation was barely noticeable within the tested concentration range, as net OCT2 uptake resembled a linear line more than a hyperbolic curve. Therefore, this compound was particularly tested with higher concentrations up to 6000 μM. As illustrated in Figure 5A, 5-amino-1-pentanol uptake by EV control cells dominates at concentrations higher than 5000 μM. This explains the reason behind the disturbance in linearity in the total uptake by EV-transfected cells.

### 2.3 Substrates Exclusive for OCT2

In order to confirm the transport activity of small aliphatic amines by OCT2, concentration-dependent experiments were conducted on a set of selected substrates with varying carbon chain lengths and substitutions, all of which exhibited a saturable uptake (Table 2, Figure 6).

**Figure 6:**
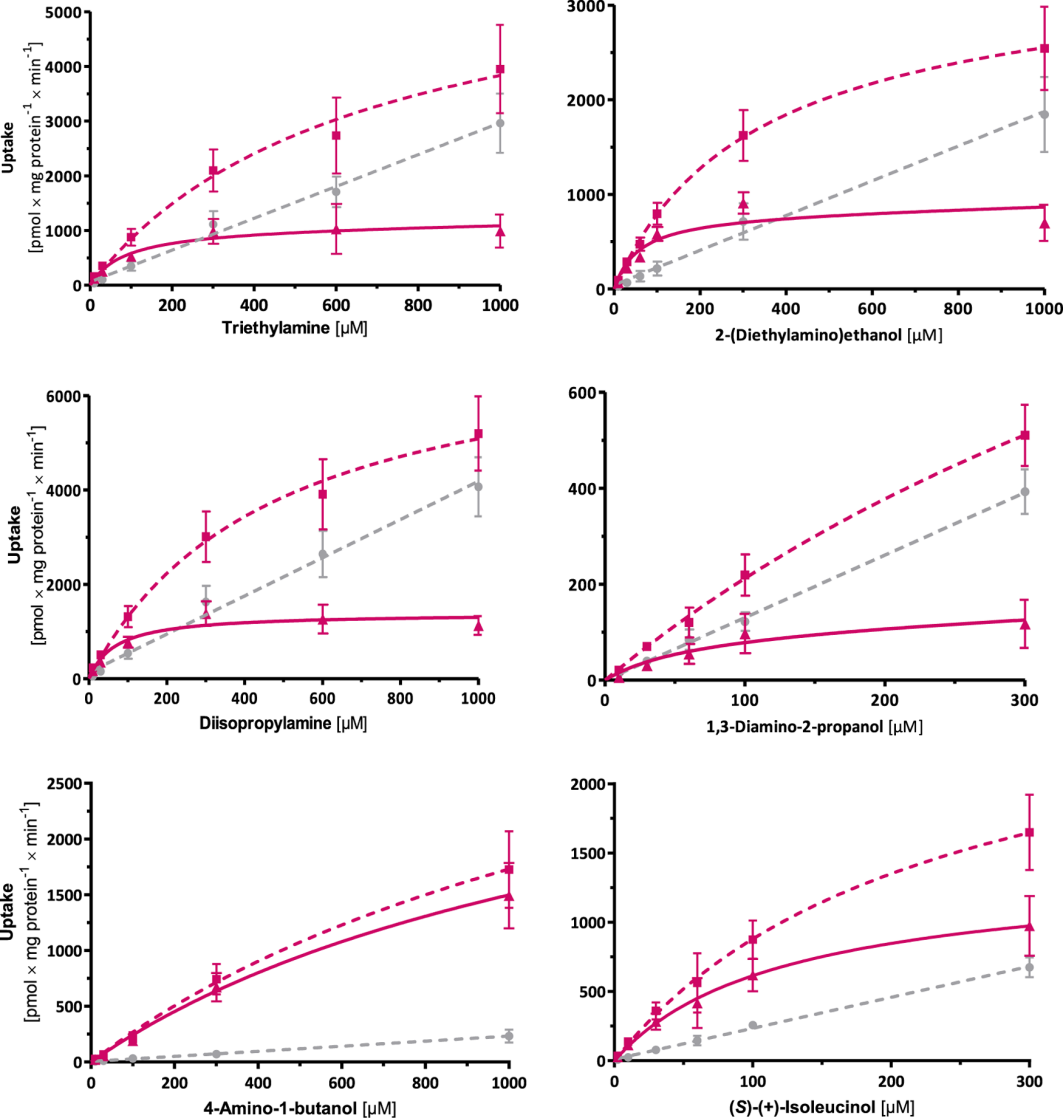
Concentration-dependent uptake of substrates exclusive for OCT2. Total uptake by OCT2 (pink-dotted curve), EV control cells (gray dotted curves), and net uptake by OCT2 (pink solid curves) are shown. Data is displayed as means ± SEM of ≥3 independent experiments.

Compounds with simpler structures of one hydroxyl group at one end of the carbon chain and one amino group at the other end, such as 4-amino-1-butanol, 5-amino-1-pentanol, and 6-amino-1-hexanol, were transported by OCT2 to a large extent but with notably low affinities (*K*_m_ > 1000 μM). Furthermore, CL_int_ values (2.5, 12, and 14 μL/mg protein/min, respectively) indicated that a longer carbon chain enhances the efficiency of transport by OCT2. This trend was observed not only for OCT2, but also for OCT1, OCT3, and MATE1. CL_int_ values consistently showed that 6-amino-1-hexanol was a better substrate than 5-amino-1-pentanol for all transporters (Table 2).

In comparison with structures where the amino and hydroxyl groups are at the end of the carbon chain, there were dramatic changes in *v*_max_ and *K*_m_ where the amino group is positioned at the second carbon atom (e.g., 2-amino-1-hexanol, 2-amino-1-pentanol, valinol, and isoleucinol). For instance, both enantiomers of 2-amino-1-hexanol had a higher affinity (considerably lower *K*_m_) for OCT2 than 6-amino-1-hexanol. Similarly, 2-amino-1-pentanol was transported by OCT2 with far higher affinity than 5-amino-1-pentanol. Moreover, extending the carbon chain of 2-aminoalcohols by one carbon atom (e.g., 2-amino-1-pentanol to 2-amino-1-hexanol) has similarly improved the affinity and thus the efficiency of OCT2-mediated transport.

Interestingly, increasing structure complexity, such as by methylation of the carbon chain in alkanolamines, further enhanced OCT2 affinity. For example, isoleucinol exhibited significantly higher affinity (*K*_m_ of 116 μM) to OCT2 than 2-amino-1-pentanol (*K*_m_ of 245 μM and 239 μM for the (*S*)- and (*R*)-enantiomer, respectively). OCT2 affinity toward different structures is illustrated in Figure 7.

**Figure 7:**
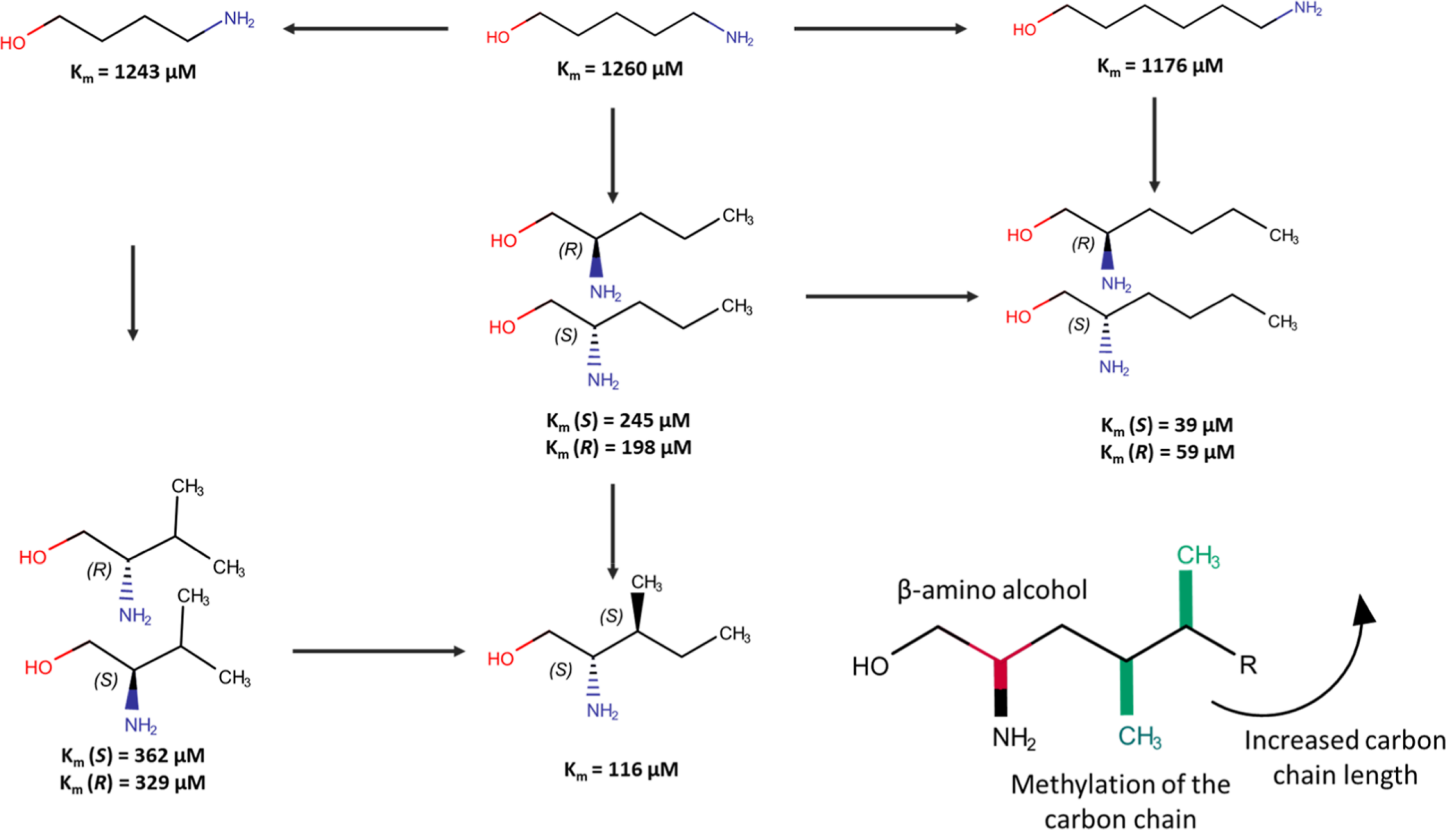
Relationships between chemical structures variations and OCT2 affinity. Arrows point toward the structure with higher affinity. Attachment of the amino group to carbon in position 2 and an increased structural complexity by elongated carbon chain lengths and carbon methylation improved the affinities for small aliphatic amines to OCT2.

Notably, OCT2-mediated transport of the secondary amine (diisopropylamine) and tertiary amines [triethylamine and 2-(diethylamino)­ethanol] with carbon chain lengths of up to three carbon atoms was characterized by high affinity transport, as all compounds exhibited *K*_m_ values of less than 100 μM. On the other hand, 1,3-diamino-2-propranol that contains two amino groups was a poor OCT2 substrate, with an intrinsic clearance of only 1.7 μL/mg protein/min. For these substances, transporter-mediated uptake prevails at low concentrations, while passive (non-ionic) diffusion becomes the dominant mode of membrane passage at high substrate concentrations (Figure 6).

To provide additional evidence for OCT2-mediated uptake of small aliphatic amines, we inhibited the transport activity of OCT2 using the potent OCT2 inhibitor imipramine (Figure 8). OCT2-mediated uptake of three substrates with different carbon chain lengths, namely diisopropylamine, (*R*)-(−)-*tert*-leucinol, and (*S*)-(+)-isoleucinol, was completely suppressed, with percent inhibition values of 112%, 128%, and 136%, respectively.

**Figure 8:**
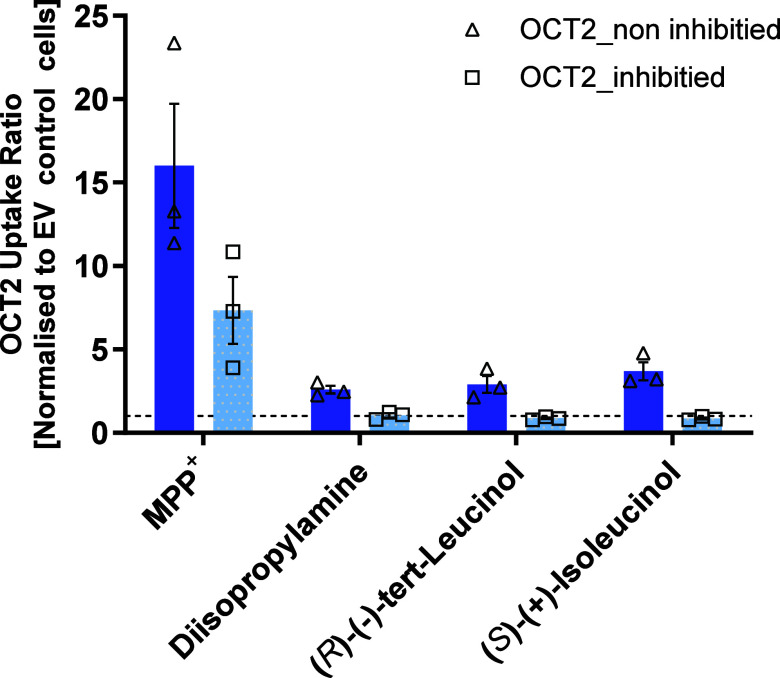
Inhibition of OCT2-mediated transport of selected substrates by imipramine. The model substrate 1-methyl-4-phenylpyridinium (MPP^+^) was used as a control to validate OCT2 inhibition. Individual data points are shown and bar charts represent the means ± SD of three independent experiments.

### 2.4 Stereoselectivity

Nine chiral aliphatic amines were tested for their transport potential by OCTs 1–3 and MATEs 1 and 2-K. Stereoselectivity was calculated by dividing the uptake ratios of the enantiomer that is transported more strongly by that of the enantiomer that showed lower transport (Table 3).

**Table 3:** Stereoselectivity in Membrane Transport Expressed as the Uptake Ratios for Individual Enantiomers of Chiral Compounds

	uptake ratios				
enantiomers	OCT1	OCT2	OCT3	MATE1	MATE2K
(*R*)-(−)-1-amino-2-propanol	1.1	2.4	0.9	1.2	1.1
(*S*)-(+)-1-amino-2-propanol	1.2	3.1	0.9	1.1	0.9
**stereoselectivity**	1.1-fold for (*S*)	1.3-fold for (*S*)		1.1-fold for (*R*)	1.3-fold for (*R*)
(*R*)-(−)-2-amino-1-propanol	1.1	1.0	1.1	1.1	1.0
(*S*)-(+)-2-amino-1-propanol	1.2	**3.3**	1.1	1.3	1.0
**stereoselectivity**	1.2-fold for (*S*)	**3.5-fold** **for**(*S*)^b^		1.2-fold for (*S*)	
(*R*)-(+)-1,2-diaminopropane	0.8	7.8	1.1	0.9	0.9
(*S*)-(−)-1,2-diaminopropane	1.1	9.5	1.5	0.9	1.0
**stereoselectivity**	1.3-fold for (*S*)	1.2-fold for (*S*)	1.4-fold for (*S*)		1.1-fold for (*S*)
(*R*)-3-aminobutan-1-ol	1.5	**7.6**	1.4	**2.1**	1.4
(*S*)-3-aminobutan-1-ol	1.4	2.8	1.7	1.1	1.0
**stereoselectivity**	1.1-fold for (*R*)	**2.7-fold** **for**(*R*)^a^	1.2-fold for (*S*)	1.9-fold **for**(*R*)^a^	1.4-fold for (*R*)
d-valinol	1.6	7.8	1.5	1.5	1.1
l-valinol	1.3	8.1	1.6	1.1	1.6
**stereoselectivity**	1.3-fold for (*R*)	1.04-fold for (*S*)	1.04-fold for (*S*)	1.4-fold for (*R*)	1.5-fold for (*S*)
(*R*)-(−)-*tert*-leucinol	1.1	7.9	1.1	0.9	1.4
l-*tert*-leucinol	1.4	10.8	**2.1**	0.7	0.9
**stereoselectivity**	1.3-fold for (*S*)	1.4-fold for (*S*)	**1.9-fold** **for**(*S*)^a^	1.3-fold for (*R*)	1.5-fold for (*R*)
(*R*)-(−)-2-amino-1-pentanol	1.1	5.0	1.0	1.6	1.8
(*S*)-(+)-2-amino-1-Pentanol	1.0	6.0	1.1	1.1	1.5
**Stereoselectivity**	1.1-fold for (*R*)	1.2-fold for (*S*)	1.1-fold for (*S*)	1.5-fold for (*R*)	1.2-fold for (*R*)
(*R*)-(−)-leucinol	1.5	8.7	1.7	1.1	1.1
(*S*)-(+)-leucinol	1.5	10.6	1.2	1.0	1.1
**stereoselectivity**		1.2-fold for (*S*)	1.4-fold for (*R*)	1.1-fold for (*R*)	
(*R*)-(−)-2-amino-1-hexanol	1.3	5.0	0.8	1.0	1.3
(*S*)-(+)-2-amino-1-hexanol	1.2	6.3	1.1	0.8	1.0
**Stereoselectivity**	1.1-fold for (*R*)	1.3-fold for (*S*)	1.3-fold for (*S*)	1.2-fold for (*R*)	1.3-fold for (*R*)

a Statistical significance of the differences between the two enantiomers using Student’s *t*-test with *p* < 0.05 and

b *p* < 0.01. Only those pairs of enantiomers in which at least one enantiomer was a substrate (uptake ratio ≥2) and that showed a statistically significant difference in transporter-mediated uptake between both enantiomers are highlighted in bold. The respective SD values of the transport ratios presented here are found in Table 1.

Strong stereoselectivity was observed with respect to 2-amino-1-propanol and l-*tert*-leucinol, as only their respective (*S*)-enantiomers were transported by OCT2 and OCT3. In contrast, MATE1 transported only the (*R*)-enantiomer of 3-aminobutan-1-ol.

Interestingly, OCT2 showed a preference for the respective (*S*)-enantiomers of its chiral substrates, except for 3-aminobutan-1-ol. An exception was 3-aminobutan-1-ol, where a 2.7-fold higher uptake ratio of the (*R*)-enantiomer over its (*S*)-enantiomer by OCT2 was observed.

To further understand stereoselectivity in OCT2-mediated transport, concentration-dependent experiments were conducted and the kinetic parameters of the two enantiomers of three chiral OCT2 substrates were compared (Table 2, Figure 9).

**Figure 9:**
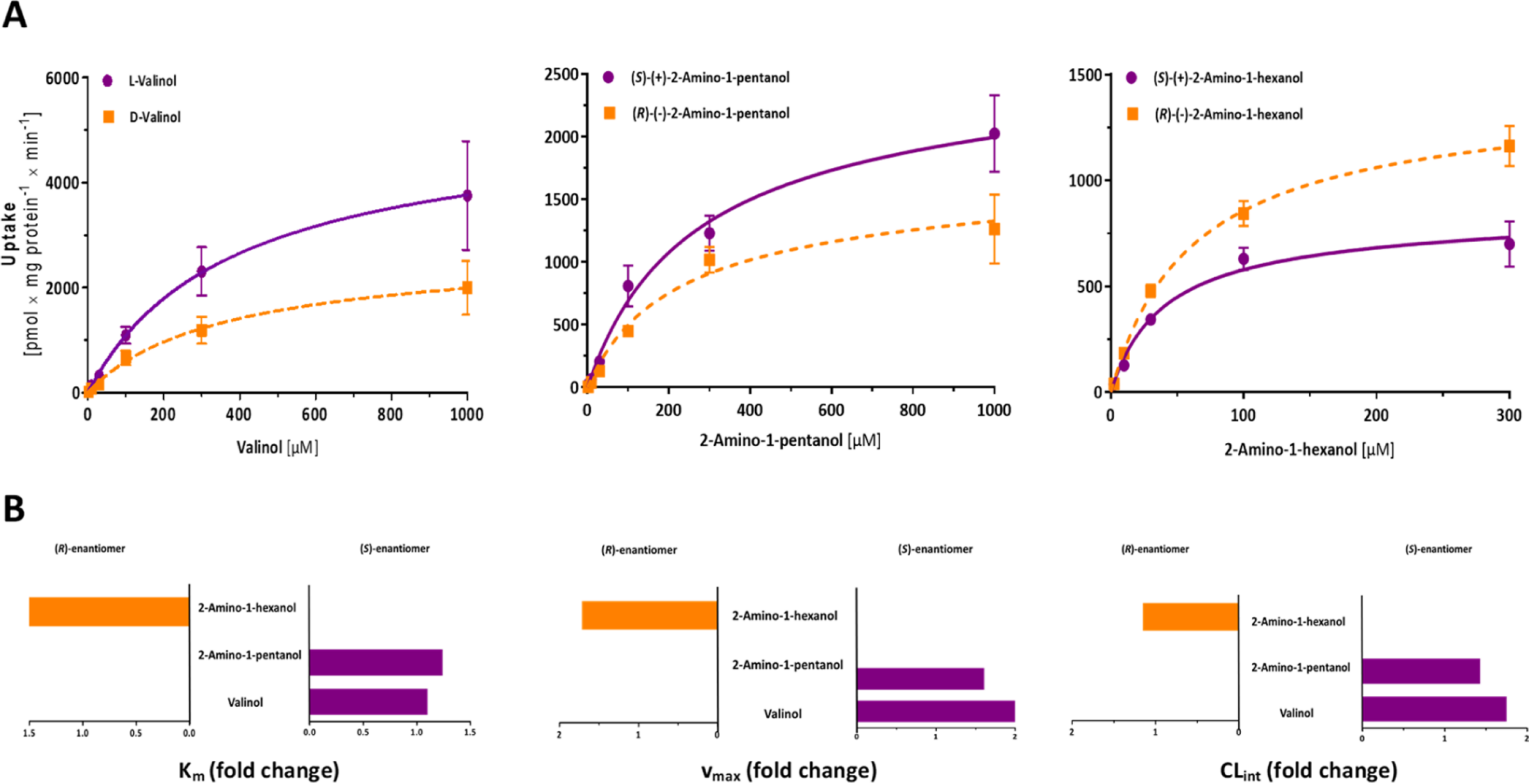
Stereoselectivity in OCT2-mediated uptake of chiral aliphatic amines. (A) Net uptake curves for both enantiomers by OCT2. Data is presented as mean ± SEM of ≥3 independent experiments. (B) Comparison of the kinetic parameters of OCT2 transport of enantiomeric compounds. Ratios were calculated as the quotients of the higher and the lower values. Purple bars on the right correspond to higher values for the (*S*)-enantiomers, while the orange bars on the left represent a larger value for the (*R*)-enantiomers.

As indicated by the uptake ratios, the (*S*)-enantiomers of valinol and 2-amino-1-pentanol were transported more efficiently and with higher capacities by OCT2 than their respective (*R*)-enantiomers. We observed a 2.0-fold and 1.6-fold higher *v*_max_ for the (*S*)-enantiomers of both compounds in comparison to their corresponding (*R*)-enantiomers.

For 2-amino-1-hexanol, the difference in CL_int_ does not suggest a notable difference in transport efficiency between its two enantiomers. However, OCT2 transported the (*R*)-enantiomer with a statistically significant 1.7-fold higher *v*_max_ (Student’s *t*-test *p* = 0.0024).

### 2.5 Analysis of Chemical Descriptors

The chemical descriptors of substrates and non-substrates were analyzed for the studied SLCs (Table 4). When comparing substrates characteristics across the investigated SLC transporters, *M*_W_ differed significantly between transporters (one-way ANOVA, *p* = 0.037). The mean *M*_W_ of OCT2 substrates (95 Da) was lower than that of substrates for the other transporters (≥113 Da; Figure 10A). OCT2 non-substrates were significantly more hydrophilic and had a higher number of hydrogen acceptors compared to the non-substrates of the other SLCs (one-way ANOVA, *p* = 0.036 and 0.024, respectively).

**Table 4:** Chemical Properties of Substrates and Non-Substrates

	chemical properties	OCT1	OCT2	OCT3	MATE1
substrates (uptake ratio ≥2)	*N* (%)	4 (8.7%)	37 (80.4%)	3 (6.5%)	4 (8.9%)
	*M*_W_ ^a^	120 (±18)	95 (±20)^c^	113 (±8)	114 (±24)
	most basic p*K*_a_	10.3 (±0.1)	10.0 (±0.4)	10.0 (±0.3)	10.2 (±0.2)
	charge at pH 7.4	1.0 (±0)	1.1 (±0.3)^d^	1.0 (±0)	1.0 (±0)
	Log *D*_pH7.4_	–2.3 (±1.0)	–2.7 (±0.7)[Table-fn t4fn5]	–2.6 (±0.7)	–3.0 (±0.5)
	TPSA	41 (±10)	39 (±13)^c^	46 (±0)	46 (±0)
	no. of hydrogen donors	1.8 (±0.5)	1.7 (±0.6)^c^	2.0 (±0)	2.0 (±0)
	no. of hydrogen acceptors	1.8 (±0.5)	1.8 (±0.5)[Table-fn t4fn5]	2.0 (±0)	2.0 (±0)
non-substrates (uptake ratio <2)	*N* (%)	42 (91.3%)	9 (19.6%)	43 (93.5%)	41 (91.1%)
	*M*_W_	99 (±27)	122 (±40)^c^	100 (±28)	99 (±27)
	most basic p*K*_a_	9.9 (±0.5)	9.8 (±0.6)	10.0 (±0.5)	10.0 (±0.5)
	charge at pH 7.4	1.3 (±0.6)	1.9 (±1.1)^d^	1.3 (±0.6)	1.3 (±0.6)
	Log *D*_pH7.4_ ^a^	–3.2 (±1.7)	–5.0 (±2.8)[Table-fn t4fn5]	–3.2 (±1.7)	–3.1 (±1.7)
	TPSA	42 (±16)	55 (±18)^c^	42 (±16)	42 (±16)
	no. of hydrogen donors	1.8 (±0.7)	2.3 (±0.7)^c^	1.8 (±0.7)	1.8 (±0.7)
	no. of hydrogen acceptors^a^	2.00 (±0.7)	2.8 (±1.0)[Table-fn t4fn5]	2.00 (±0.8)	1.9 (±0.8)

a Descriptors were tested for statistical significance between transporters using one-way ANOVA (*p* ≤ 0.05).

b Statistical differences in descriptors between substrates and non-substrates within the same transporter were also tested using Student’s *t*-test with *p* ≤ 0.01,

c *p* ≤ 0.001, and

d *p* ≤ 0.0001.

**Figure 10:**
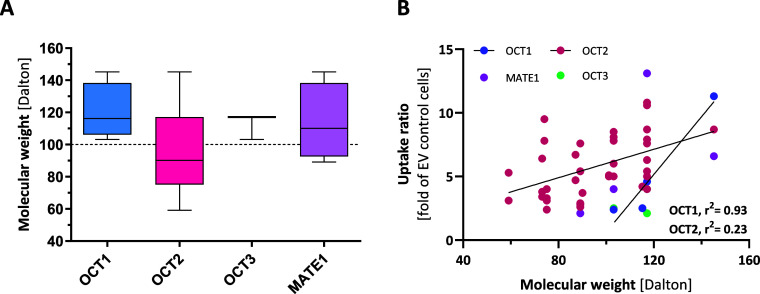
Potential influence of *M*_W_ on transport potential by different SLCs. (A) Distribution of *M*_W_ for substrates of OCTs 1–3 and MATE1, defined by an uptake ratio ≥2. (B) Correlation between uptake ratios and substrate *M*_W_ for OCT1 (blue), OCT2 (red), OCT3 (green), and MATE1 (pink).

A positive correlation between substrate *M*_W_ and uptake ratio were observed (Pearson *r* = 0.4, *p* = 0.005), and linear regression indicated that 16% of the variation in uptake is explained by the substrate *M*_W_ (*r* ^2^ = 0.16, *p* = 0.005, Figure 10B). The transport activities of OCT1 and OCT2, in particular, are increased with higher *M*_W_ (*r* ^2^ = 0.93, *p* = 0.04, *n* = 4 for OCT1 and *r* ^2^ = 0.23, *p* = 0.003, *n* = 37 for OCT2).

Furthermore, within-transporter analyses showed that OCT2 substrates had smaller *M*_W_ (Student’s *t*-test, *p* = 0.007) and smaller topological polar surface area (TPSA, *p* = 0.004) compared to non-OCT2 substrates. Substrates were also less positively charged (*p* = 0.0001) and less hydrophilic (*p* < 0.0001) than non-substrates. Additionally, the number of hydrogen donors and acceptors were significantly lower in OCT2 substrates than in non-substrates (*p* = 0.005 and *p* < 0.0001, respectively).

## 3 Discussion

In this study, we sought to systematically explore the relationship between the chemical structure of simple aliphatic organic molecules and their properties as substrates of OCTs and MATEs. To this end, we have selected test compounds with simple positively charged structures in order to gain insight into the structure–function relationships of SLCs. Given the (approximately 70%) high sequence homology between human OCTs, a large overlap in substrate specificity has frequently been reported.^1,10,12^ Our results, however, showed an exceptionally broad OCT2 substrate profile, a surprisingly low number of substrates among our set of aliphatic amines for OCT1, OCT3, and MATE1, and none at all for MATE2-K. OCT2 has consistently demonstrated a more promiscuous behavior and broader substrate spectrum compared to OCT1 and OCT3. Even with previously identified shared substances, OCT2 showed stronger transport than OCT1 and OCT3.^10,13,17^

### 3.1 Structure–Activity Relationship Analysis

Main findings include the observation that OCT1, OCT3, and MATE1 showed no transport activity with molecules that have carbon chain lengths of fewer than five carbon atoms. Only OCT2 was capable of transporting compounds with carbon chain lengths as short as two or three atoms. Consistent with our findings, a recent cryogenic electron microscopy structure of OCT1 revealed that a net positive charge and relatively high lipophilicity are integral to substrate recognition and translocation by OCT1.^6^ The critical novel role of the acidic residue E386 (TM8) in electrostatic interactions with the positively charged nitrogen of OCT1 substrates was specifically highlighted. Additionally, four hydrophobic aromatic and aliphatic residues, namely W217, F244, W354, and I446, play a key role in stabilizing the substrate within the central cavity of the transporter’s binding pocket.^6^ A lack of transport activity for small aliphatic amines by OCT1, OCT3, MATE1 and MATE2-K could be attributed to insufficient hydrophobic interactions with these compounds, which possibly hinders effective substrate stabilization within the binding pocket. This is supported by our observation that the elongation of the carbon chain enhanced affinity and overall translocation efficiency. The distinct behavior of OCT2, on the other hand, could possibly be due to the presence of an additional negatively charged residue, E448, in proximity to E387.^6^ This feature enhances cation binding by increasing the electronegative surface potential in this region of OCT2’s central cavity. Consequently, this might improve substrate stabilization, subsequent isomerization and transport efficiency, even for smaller and less lipophilic aliphatic amines. On the contrary, Zhang et al. (1999) demonstrated that increasing the alkyl chain length of *n*-tetraalkylammonium compounds—and thereby lipophilicity—reduced the rate of transport by OCT1 but increased their inhibition potencies.^18^ However, these compounds have quaternary ammonium structures with a permanent positive charge, and their binding properties to OCTs might thus substantially differ.

While the hydrophilic/hydrophobic nature of a substance likely influences the transport potential by OCTs, substrate selectivity appears to be more complex and cannot be fully explained by physicochemical properties alone. This is supported by our observation that only structures, in which the amino and hydroxyl groups are separated by at least five carbon atoms, functioned as substrates for OCT1, OCT3, and MATE1. In contrast, structurally analogous aliphatic amines with nearly identical chemical characteristics but differing positions of the amino group were not transported by these transporters. Conversely, OCT2 transported compounds with a diverse range of structures where the amino group was attached to the second, third, or terminal carbon atom. This may be attributed to hydrogen bond formation with Y362 (TM7) that is in proximity to the additional acidic residue E448 (TM10) exclusive to OCT2.^6^ Interestingly, the amino group position dramatically influenced the kinetic parameters, particularly substrate affinity. The transport of compounds with one amino group at one end and one hydroxyl group at the other end was characterized by a particularly low affinity (*K*_m_ > 1000 μM), which was reflected in a barely noticeable saturation within the tested concentration range (Figure 5). Testing higher concentrations is needed to observe the hyperbolic behavior of the transport curve. Nevertheless, this poses another issue of artifacts that limit precise quantification. On the other hand, substrates with β-amino alcohol structure, in which the amino and hydroxyl groups are separated by a single carbon atom, demonstrated a higher affinity. The chiral aliphatic amine 2-amino-1-hexanol, characterized by a six-carbon chain and a β-amino alcohol structure, was by far the most efficient substrate for OCT2, exhibiting the highest affinity and intrinsic clearance value (Table 2). Notably, increasing the structure’s complexity through methylation of the carbon chain also improved OCT2 affinity and transport activity, as exemplified by the comparison between isoleucinol and 2-amino-1-pentanol.

### 3.2 Other Identified Substrates Among Aliphatic Amines

The vast majority of previously identified OCT substrates are relatively rigid compounds containing at least one aromatic ring. A notable exception is the antituberculosis drug ethambutol, which features an aliphatic amine structure similar to those tested in this study. It has been reported to be a good substrate for several transporters for organic cations, including OCTs 1–3 and MATEs 1 and 2-K.^10,19^ Other substrates with aliphatic structures include guanidine the antidiabetic drug metformin (*N*,*N*-dimethylbiguanide). The quaternary amines choline, acetylcholine, and the prototypical substrate tetraethylammonium are also well-known substrates of various OCTs and MATEs.^1^

### 3.3 Molecular Weight as an Important Driver for SLC-Mediated Transport

It is well established that the substrates of OCTs typically have a *M*_W_ below 500 Da.^3,11,12^ In our study, we found a lower limit for substrate *M*_W_. Except for OCT2, a minimum *M*_W_ of 100 Da appears to be a prerequisite for substrate recognition by all SLCs studied here. Correspondingly, a strong correlation between cellular uptake and substrate *M*_W_ was observed, particularly for OCT1 and OCT2. Notably, OCT1 substrates have previously been reported to exhibit a higher *M*_W_ than substrates of OCT2 and OCT3.^10^ Our data fits into the findings of Hendrickx et al. (2013), who also found increased OCT1-mediated uptake with higher *M*_W_ up to 300 Da for a specific drug class containing a β-amino alcohol moiety.^12^ However, their study did not include molecules with *M*_W_ below 100 Da.

To the best of our knowledge, the transport potential of small substances (*M*_W_ < 100 Da) by OCTs has not been investigated thoroughly before. The majority of previous studies have rather focused on the role of OCTs in the transport of clinically used small-molecule drugs, which typically have a *M*_W_ exceeding 150 Da. Our observation that OCT2 is the only organic cation transporter to exhibit activity with very small compounds suggests that it possesses distinctive, yet unrecognized biological functions in the translocation of diminutive positively charged molecules. Although many endogenous substrates have been identified as substrates of OCTs, the physiological roles of OCTs remain incompletely understood. According to the Human Metabolome Database (HMDB), there are at least 490 human metabolites classified as organic cations with at least one positive charge and a *M*_W_ below 150 Da.^20^ Interestingly, not much is known about the transport of these molecules across biological membranes. Since these substances are hydrophilic and positively charged at physiological pH, non-ionic diffusion would contribute to their translocation only to a limited extent. Hypothetically, OCT2 might contribute to the renal elimination of such substances. This idea is supported by the fact that, in uremia, there is an accumulation of several short chain aliphatic amines, such as dimethylamine and trimethylamine, contributing to various uremic symptoms.^21^

No cases of inherited complete OCT2 deficiency have been reported in humans. However, combined OCT1 and −2 knockout mice were studied on that question, and changes in blood concentrations of several low molecular weight organic cations have been observed.^22^

### 3.4 OCT2 as an Ethanolamine Efflux Transporter

Ethanolamine is a simple organic molecule with amino and hydroxyl groups positioned at opposite ends (Figure 1). It is considered essential to life due to its role as a building block for phospholipids. Ethanolamine is involved in the synthesis of phosphatidylethanolamine and other lipids via the Kennedy pathway.^23^ To exert its biological function, ethanolamine must be transported into the cell. However, its transport pathway remains poorly understood, with only one study suggesting the involvement of choline transporter-like proteins 1 and 2 (*SLC44A1* and *SLC44A2*) as ethanolamine uptake transporters.^24^

Intriguingly, some compounds in our study, including ethanolamine, appeared to be transported out of the transporter-transfected cells. In OCT1- and OCT2-overexpressing cells, ethanolamine uptake was reduced to approximately one-third and two-thirds, respectively, of the uptake observed in EV control cells. This phenomenon is most plausibly explained by an influx of ethanolamine, mediated either by passive, non-ionic diffusion or by other transporters constitutively expressed in the HEK293 cells, followed by OCT1- and OCT2-mediated efflux, resulting in an uptake ratio significantly below 1 (Figure 2, Table 1). Notably, an efflux function of OCT1 with acylcarnitine has been previously reported.^25^ Therefore, OCT1 and, particularly OCT2, might be involved in the regulation of intracellular ethanolamine concentrations.

### 3.5 Polyamines and Positive Net Charge

Polyamines have at least two amino groups that are positively charged at physiological pH. Some naturally occurring polyamines, such as the spermidine, spermine, and putrescine play significant roles in mammalian cell metabolism.^26^ Understanding the homeostasis and underlying metabolic pathways of these molecules has received considerable interest due to their potential implication in various human diseases, including cancer and central nervous system pathologies. Therefore, targeting the so-called and yet not fully characterized “polyamine transport system” was suggested as a promising therapeutic strategy to treat cancer.^27^

Here, we examined the transport potential of various structurally analogous polyamines by SLCs. Interestingly, from all polyamines studied here, only 1,2-diaminopropane, which contains two amino groups, was an OCT2 substrate. In contrast, neither the biogenic spermidine, 3,3′-diamino-*N*-methyldipropylamine, tris­(2-aminoethyl)­amine, nor tris­(3-aminopropyl)­amine were transported by any of the studied SLCs. All of them have at least two primary and one secondary or tertiary amino groups.

Our results contradict those of Sala-Rabanal et al. (2013), who found that spermidine is a low-affinity substrate for OCTs 1–3, with a high *K*_m_ value of 1 mM. A modest (up to 3-fold) increase in spermidine uptake by OCT-expressing oocytes compared to non-injected oocytes was observed.^28^ However, it should be noted that these data were obtained using rodent orthologues rather than human OCTs. Interspecies differences in transport kinetics and, in some cases, even transport selectivity exists for some compounds.^29–31^ Spermidine was also reported to be a substrate for OCT2 with very low affinity (*K*_m_ = 6.76 mM) by Higashi et al. (2014).^32^ Lack of transport activity in our study could be attributed to the use of spermidine at very low concentrations (2.5 μM) compared to the 500 μM used by these authors. Other polyamines, such as putrescine and the polyamine precursor agmatine, have also been identified as OCT2 and MATE1 substrates.^33^

Although many SLCs have been suggested to contribute to the cellular translocation of polyamines, their involvement remains only putative. To date, only SLC18B1 and OCTN1 are well-acknowledged as established transporters for biogenic polyamines.^26^

Our findings suggest that compounds containing more than two amino groups are not transported by OCTs 1–3 and MATE1 and 2-K. However, it is not simply a question of the number of nitrogen atoms. For instance, the antidiabetic drug metformin with its five nitrogen atoms is a well-established substrate of most OCTs.^1^ The difference lies in the structural arrangement. In OCT substrates carrying one or two guanidine groups, the positive charge is concentrated on one or two molecular sites, whereas in our not-transported polyamines, there is a spacing formed by several carbon atoms and positive charge is distributed across at least three nitrogen atoms (Figure 1).

It has been well established that a positive charge is required for substrate recognition by OCTs.^11^ In fact, monovalent cations constitute the vast majority of substances that interact with OCTs. Divalent cations, have also been reported to be OCT substrates.^1^ However, it remains unclear whether substances with a net physiological charge of +3 or higher are translocated across cellular membranes via SLCs. As suggested by the cryogenic electron microscopy structures, the presence of two acidic residues in OCT1 and three in OCT2 in the orthosteric site offer possible sites for electrostatic interactions with divalent and trivalent cations. A simple explanation for the fact that they are not transported might be that they bind too strongly to the negatively charged amino acids in these transporters. Strong bond formations could obstruct the necessary conversion from the outward-facing open to the outward-facing occluded state, and thus inhibitory effects might be observed. In fact, polyamines have been previously reported to inhibit OCTs.^34^

Interestingly, OCT2-mediated uptake of agmatine, putrescine, and spermidine was enhanced under more alkaline conditions.^32,33^ The proportions of monovalent, divalent, and trivalent forms of these compounds are pH-dependent, with the relative concentrations of lower-valent forms increasing as pH rises. While pH has also influenced the uptake of permanently charged compounds, such as quaternary amines,^33^ and other underlying mechanisms might be involved, the ionization state of these compounds likely plays a key role. This observation aligns with our findings that the net charge is inversely correlated with OCT transport activity.

### 3.6 Stereoselectivity in SLC-Mediated Transport

Stereoselectivity in membrane transport has been previously reported^35,36^ and, more recently, has been extensively studied in the context of SLCs.^13–15^ In the present study, we examined the extent of stereoselectivity in SLC-mediated transport of aliphatic amines. Notably, where stereoselectivity was observed, OCT2 showed a preference for the respective (*S*)-enantiomers for all but one of the chiral substrates. Despite the observed low transport activity of small aliphatic amines by the SLCs other than OCT2, OCT3 and MATE1 exhibited enantiospecific uptake of certain compounds, showing no noticeable uptake of one of the respective enantiomers. OCT1, however, transported all enantiomers of chiral substances with comparable activity, thereby showing little to no stereoselectivity. Our results are in accordance with previous reports, which showed that stereoselectivity is more pronounced with OCT2 and OCT3 compared to OCT1.^13,15^ The limited overall stereoselectivity observed here could be attributed to the structural flexibility of the studied aliphatic amines, which allows for multiple configurations within the transporter binding pocket. This is in agreement with previous observations of low stereoselective uptake of the aliphatic drug ethambutol among a set of chiral drugs.^15^

Interestingly, only 2-amino-1-propanol but not its constitutional isomer 1-amino-2-propanol showed stereoselective interaction with OCT2, as OCT2 interacted similarly with both enantiomers of 1-amino-2-propanol (Figures 1 and 2, Table 1). This finding aligns with that of Gross and Somogyi (1994). By studying the stereoisomers of noradrenaline and adrenaline, they concluded that stereoselective uptake by OCTs occurs only with enantiomers, where the center of chirality is located on the carbon atom adjacent to the nitrogen functional group.^37^

### 3.7 Analytical Method

Membrane transport studies often utilize fluorogenic or radiolabeled substrates. However, the substrates investigated here are non-fluorogenic, and synthesizing radiolabeled forms would have incurred significant costs and required extensive efforts for synthesis and purification. To overcome these challenges, we initially attempted to analyze all substrates using reversed-phase high-performance liquid chromatography coupled with tandem mass spectrometry (HPLC-MS/MS). However, several substrates exhibited poor retention on the columns, even without solvent additives in the eluent. Consequently, they eluted almost immediately after the void volume, leading to low sensitivity and other types of interferences that could not be resolved by selecting alternative fragment masses. Next, we explored hydrophilic interaction liquid chromatography (HILIC) columns recommended in this situation (details not shown). While this approach allowed detection and quantification of the pure substances, the resolution was insufficient with the complex cell lysates, resulting again in low sensitivity and interferences. To address these limitations, we ultimately adopted derivatization with the fluorogenic 6-aminoquinolyl-*N*-hydroxysuccinimidyl carbamate (AQC), followed by analysis using LC–MS/MS, as detailed in the methods section. While AQC derivatization also enables fluorescence detection, the presence of numerous amines in the cell lysate complicated separation of analytes from other lysate components.

Another type of challenge always arises when measuring the transport of substances endogenously present in biological samples, such as ethanolamine and spermidine. To mitigate interference from endogenous ethanolamine and spermidine, we here employed ^13^C_2_-ethanolamine and deuterium-labeled spermidine isotopes as substrates, which could be specifically identified by tandem mass spectrometry.

### 3.8 Conclusions

To summarize, our data suggests that a minimum *M*_W_ of 100 Da is required for efficient OCT1-, OCT3-, MATE1-, and MATE2-K-mediated uptake, with the notable exception of OCT2. Small hydrophilic amines appear to be preferentially, and perhaps exclusively, transported by OCT2. Biologically, such substances may arise from nutritional sources, from endogenous metabolism, and from the intestinal microbiome. Examples for that were already highlighted early in research on OCT2.^22^ However, thus far, most substances identified as substrates of OCT2 had at least one aromatic or aliphatic ring structure and thus the broad range of aliphatic substrates was unexpected. Moreover, our findings underscore the importance of substrate structural complexity in determining the transport activity of SLCs. While general physicochemical features and a size cut-off approach provide a useful framework for predicting potential substrates, distinct molecular features are essential for efficient transport. Even minor modifications to the chemical structure of aliphatic amines can lead to a complete loss of transport or cause significant alterations in affinity. Additionally, increasing the positive net charge above +2 with the presence of more than two basic functional groups results in complete lack of transport potential by any of the studied SLCs. This study further confirms the previously reported modest extent of stereoselectivity in SLC-mediated transport, here even at a lower *M*_W_ below 100 Da. Altogether, our findings provide new insights into substrate preferences of polyspecific SLCs. The molecular features of OCT substrates elucidated in our study can be implemented as valuable tools in the drug design process, enabling the development of compounds with improved hepatic and renal elimination, and consequently enhanced pharmacokinetics profiles.

## 4 Material and Methods

### 4.1 Test Compounds

In total, a set of 46 aliphatic amines was investigated for transport potential by OCTs and MATEs (Table S1). To avoid interference with substances that also exist in our model cell line endogenously, the respective isotopes, ethanolamine-^13^C_2_ and deuterated spermidine, were used. (*R*)-(−)-2-amino-1-hexanol and (*S*)-(−)-leucinol were purchased from Santa-Cruz Biotechnology (Dallas, Texas, USA), (*R*)-*tert*-leucinol from Thermo Fisher Scientific (Darmstadt, Germany), and all other tested aliphatic amines from Sigma-Aldrich Chemie GmbH (Darmstadt, Germany). According to the manufacturers, the purities of all compounds were at least 95%, except for 5-amino-1-pentanol, which was commercially available only at a purity of 92%.

### 4.2 In Vitro Cellular Uptake Experiments

Transport assays were conducted using HEK293 cells stably transfected by genomic integration of the respective cDNA to overexpress the respective human transporter of interest: OCT1 (*SLC22A1*), OCT2 (*SLC22A2*), OCT3 (*SLC22A3*), MATE1 (*SLC47A1*), or MATE2-K (*SLC47A2*). The protein MATE2-K as a functional splice variant lacking 36 amino acids in the loop between the fourth and fifth transmembrane domains (TM) of MATE2.^38^ Cells transfected with the EV pcDNA5 served as controls to account for non-transporter-specific transport (non-ionic diffusion). All cell lines were generated using the Flp-In system (Thermo Fisher Scientific, Darmstadt, Germany), as previously described.^39^

Cells were cultivated in Dulbecco’s modified Eagle’s medium supplemented with 10% (v/v) fetal bovine serum and 1% (v/v) penicillin (100 U/mL)/streptomycin (100 μg/mL). Forty-eight h prior to the transport experiments, 600,000 or 300,000 cells were seeded into poly-d-lysine precoated 12- or 24-well plates, respectively, and incubated at 37 °C, 95% relative humidity, and 5% CO_2_. Cells were discarded after a maximum of 35 passages in culture.

On the day of the experiment, cells were initially washed with prewarmed (37 °C) HBSS (Hanks’ Balanced Salt Solution, Thermo Fisher Scientific, Darmstadt, Germany) supplemented with 10 mM HEPES (Sigma-Aldrich, Darmstadt, Germany), henceforth referred to as HBSS^+^, to maintain a physiological pH of 7.4. Since MATE1 and MATE2-K are proton antiporters responsible for exporting organic cations from the cells to the extracellular space with higher proton concentration (e.g., the tubules in kidneys), a 30 min preincubation with 30 mM ammonium chloride resulting in intracellular acidification was used to reverse the transport direction.^16^ Afterward, cells were incubated with the test substance for one or two min for MATEs- or OCTs-overexpressing cells, respectively, on a heating block prewarmed to 37 °C. The intracellular acidification dissipates a few minutes after withdrawal of the 30 mM NH_4_Cl. Therefore, a shorter incubation time of 1 min was chosen for the MATEs. The incubation was stopped by adding ice-cold HBSS^+^ to each well, followed by two washing steps. The cells were then lysed with 80% (v/v) acetonitrile/water containing a fixed concentration of an analytical internal standard (IS). Intracellular uptake was determined using HPLC-MS/MS.

For total protein quantification per well, two additional wells per cell line were lysed using radioimmunoprecipitation assay buffer (50 mM Tris HCl pH 7.4, 150 mM sodium chloride, 1.0 mM EDTA, 1% Nonidet P-40, 0.25% sodium deoxycholate, and 0.1% sodium dodecyl sulfate), and a standard bicinchoninic acid assay was performed.^40^ All cellular uptake data were subsequently normalized to the protein concentration.

### 4.3 Quantification of Substrate Concentrations

The intracellular concentrations of transported test compounds were determined by HPLC-MS/MS using a Shimadzu Nexera HPLC system consisting of an LC-30AD pump equipped with a SIL-30 AC autosampler, a CTO-20AC column oven, and a CBM-20A controller (Shimadzu, Kyoto, Japan). HPLC separation was achieved through reversed-phase liquid chromatography, operating at 40 °C with a flow rate from 300 to 400 μL/min. The aqueous mobile phase contained 3%, 8%, or 20% organic additive of acetonitrile/methanol 6:1 (v/v) and 0.1% (v/v) formic acid. The stationary phase comprised a Brownlee SPP RP-Amide column (4.6 × 100 mm inner dimension and 2.7 μm particle size), fitted with a Phenomenex C-18 precolumn. Analytes were consequently detected with an API 4000 tandem mass spectrometer (AB SCIEX, Darmstadt, Germany) operating in multiple-reaction-monitoring mode. Concentrations were calculated using the software Analyst (AB SCIEX, version 1.6.2). A comprehensive description of HPLC conditions and mass spectrometric detection parameters is provided in Table S2.

To ensure specificity of quantification by LC–MS/MS, blank lysates were analyzed using the same acquisition method as the test substance. Subsequently, mass transitions (Q1/Q3) that provided the highest specificity, sensitivity, and resolution were selected as quantifiers and qualifiers for LCMS/MS analyses.

For calculating cellular uptake ratios, substances were tested at a single concentration of 2.5 μM. If the signal was too low for mass spectrometric detectability, a concentration of 10 μM was used instead (indicated in Table 1). For concentration-dependent analyses, serial dilutions were performed to incubate cells with increasing concentrations of the test compound. Absolute concentrations of substrate transported into the cells were determined in reference to a standard curve generated from known concentrations of the respective compound.

### 4.4 Derivatisation

The mass spectrometric analysis of several analytes was compromised by a significant matrix effect, indicated by a significant ion suppression and a complete loss of ion intensity of the target analytes (isopropylamine, ethanolamine-^13^C_2_, *tert*-butylamine, 3-amino-1-propanol, (*R*)- and (*S*)-2-amino-1-propanol, (*R*)- and (*S*)-1-amino-2-propanol, and tris­[2-aminoethyl]­amine). Additionally, the blank cell lysate exhibited matrix interferences with all possible (Q1/Q3) mass transitions for some test substances (5-amino-1-pentanol, (*R*)- and (*S*)-1,2-diaminopropane, 3,3′-diamino-*N*-methyldipropylamin, and tris­[3-aminopropyl]­amine). To overcome these detection issues, a precolumn derivatization using AQC (Cayman Chemical, Ann Arbor, Michigan, USA; product number, 30,877) was performed.

Primary and secondary amines react with AQC, yielding highly stable unsymmetrical urea derivatives with favorable reversed-phase chromatographic properties. A 10.5 mM AQC solution was prepared by dissolving 3 mg of AQC in 1 mL of acetonitrile and heating at 55 °C for no longer than 15 min to ensure complete solubilization.

For the derivatization reaction, 10 μL of the sample was mixed with 70 μL of borate buffer (0.2 M sodium borate, pH 8.8). Subsequently, 20 μL of AQC solution was added, followed by another vortex mixing step, and the mixture was heated at 55 °C for 10 min.

Due to the non-volatility and potential problems pertaining to the mass spectrometric detection arising from the use of borate buffer,^41^ liquid–liquid extraction of the derivatized product using ethyl acetate as the organic solvent was performed. Eventually, samples were dried under nitrogen gas at 40 °C and reconstituted in 0.1% formic acid.

Direct infusion of individual derivatives was carried out to set parameters for electrospray ionization mass spectrometric detections. The derivatization reaction generated a common daughter ion at 171 *m*/*z*, resulting from the loss of the aminoquinoline moiety. Due to its high abundance, this ion was used for subsequent quantification of most derivatized analytes. For compounds with two derivatization sites (polyamines), all possible products were inspected. However, doubly and triply derivatized products represented the most abundant case.

The chemical structures of the possible derivatization products along with their *M*_W_ is presented in Figure S1. A detailed description of the chromatographic and mass spectrometric settings for the detection of derivatized aliphatic amines are found in Table S2.

### 4.5 In Vitro Inhibition Experiments

Similarly to the uptake experiments, 300,000 OCT2-transfected HEK293 cells per well were plated in 24-well plates and incubated for 2 days before the assay. Subsequently, cells were incubated with 2.5 μM of the substrate-in-question with or without 25 μM imipramine as a potent OCT2 inhibitor for 5 min^42^ To increase the signal of the test substances, and thereby a higher precision of their quantification, a longer incubation time for the inhibition experiments is needed. For each substrate, one additional well of EV-transfected cells was used as negative control to account for substrate entering the cells by non-ionic diffusion.

### 4.6 Calculations

Uptake ratios of tested substances into transporter-overexpressing cell lines were calculated as a fold-change relative to uptake into EV control cells. An uptake ratio of at least 2 was considered notable transport. This threshold value was recommended by the U.S. Food and Drug Administration, and has proved previously to effectively distinguish between substrates and non-substrates.^11^

Transporter-mediated net transport was estimated by subtracting the total uptake into EV-transfected control cells from the total uptake measured in transporter-transfected cells. Kinetic parameters, including *v*_max_ and the Michaelis constant (*K*_m_, defined as the substrate concentration [*S*] at half *v*_max_), were calculated by non-linear regression using the Michaelis–Menten equation: {*v* = *v*_max_ × [*S*]/(*K*_m_ + [*S*])}. CL_int_ was subsequently calculated as the ratio of *v*_max_ over *K*_m_. Intrinsic clearance values were used to categorize substrates into good (>5 μL/mg protein/min), poor (1–5 μL/mg protein/min), or non-substrates (<1 μL/mg protein/min). All transport and inhibition data were generated from at least three independent experiments performed at different days.

The percentage of OCT2 inhibition was calculated according to the following equation

$$\%\;OCT2\;inhibition=100\%-\frac{\left[{substrate}_{inhibited}\right]-\left[{substrate}_{passive\;diffusion}\right]}{\left[{substrate}_{non‐inhibited}\right]-\left[{substrate}_{passive\;diffusion}\right]}$$

Chemical structures were drawn and physicochemical properties, including p*K*_a_, Log *D*_pH7.4_, and percentage of positively charged compound at pH 7.4, were predicted using MarvinSketch^®^ (version 24.1.3; Chemaxon, Budapest, Hungary). GraphPad Prism 5.01 (GraphPad Software, La Jolla, CA, United States) was used for statistical analyses, and a *p*-value of less than 0.05 was considered statistically significant.

## Supplementary Material



## Notes

Table S1: physicochemical properties of tested aliphatic amines. Table S2: HPLC and mass spectrometric detection parameters of investigated aliphatic amines. Figure S1: amines subjected to the derivatization reaction and all of their possible derivatization products, along with the *M*_W_ before and after the derivatization reaction. Chiral centers are marked with an asterisk. Structures were depicted and the *M*_W_ calculated using MarvinSketch^®^ (version 24.1.3; Chemaxon, Budapest, Hungary). (PDF)

## Abbreviations

AQC: 6-aminoquinolyl-*N*-hydroxysuccinimidyl carbamate
CE: collision energy
CL_int_: intrinsic clearance
CXP: collision cell exit potential
DP: declustering potential
EV: empty vector
HBSS: Hanks’ Balanced Salt Solution
HPLC-MS/MS: high-performance liquid chromatography coupled with tandem mass spectrometry
IS: internal standard
*K*_m_: Michaelis constant
MATE: multidrug and toxin extrusion protein
*M*_W_: molecular weight
OCT: organic cation transporter
Q1: first quadrupole
Q3: third quadrupole
RT: retention time
SLC: solute carrier
TM: transmembrane domain
TPSA: topological polar surface area
*v*_max_: maximum transport velocity
