# Supplementary material for: Transport of Small Aliphatic Amines by Polyspecific Solute Carriers: Deciphering Structure–Function Relationships
Source: ACS Pharmacol Transl Sci. 2025 Jul 16;8(8):2777–94. doi: 10.1021/acsptsci.5c00340 (PMC12340637; doi:10.1021/acsptsci.5c00340)
Supplement: Supplementary file 1 [file pt5c00340_si_001.pdf]

## Supporting Information

# Transport of small aliphatic amines by polyspecific solute carriers:

# Deciphering structure-function relationships

Wouroud Ismail Al-Khalil[a], Jürgen Brockmöller[a], and Muhammad Rafehi[a,*]

[a] *Institute of Clinical Pharmacology, University Medical Center Göttingen, D-37075 Göttingen, Germany*

*Corresponding author at: Institute of Clinical Pharmacology, University Medical Center Göttingen, Robert-Koch-Str. 40, 37075 Göttingen, Germany.

E-mail address: muhammad.rafehi@med.uni-goettingen.de

## Table of Contents

**Table S1.** Physicochemical properties of tested aliphatic amines S1 **S2**

**Table S2.** HPLC and mass spectrometric detection parameters of investigated aliphatic amines **S3**

**Figure S1.** Amines subjected to the derivatisation reaction and all of their possible derivatisation products, along with the molecular weight before and after the derivatisation reaction. **S5**

**Table S1. Physicochemical properties of tested aliphatic amines**

| Substance | MW (Da) | $pKa_{basic}$ | Positive charge at pH 7.4 (%) | | $LogD_{pH\ 7.4}$ |
|---|---|---|---|---|---|
| Ethanolamine-$^{13}C_2$ | 63.07 | 9.55 | 99.3 | + | -3.49 |
| Triethylamine | 101.19 | 10.81 | 99.96 | + | -2.08 |
| Diethanolamine | 105.14 | 8.86 | 96.63 | + | -3.16 |
| Dimethylaminoethanol | 89.14 | 9.03 | 97.7 | + | -3.49 |
| *N*-Methyldiethanolamine | 119.164 | 8.7 | 95.18 | + | -2.65 |
| 2-(Diethylamino)ethanol | 117.19 | 9.55 | 99.3 | + | -2.05 |
| Tris(2-aminoethyl)amine | 146.23 | 9.85 | 99.94 | +++ (96.85) | -8.1 |
| Propylamine | 59.11 | 10.21 | 99.85 | + | -2.52 |
| Isopropylamine | 59.11 | 10.43 | 99.91 | + | -2.75 |
| Diisopropylamine | 101.19 | 11.32 | 99.99 | + | -2.13 |
| 3-Amino-1-propanol | 75.11 | 9.84 | 99.64 | + | -3.7 |
| (*R*)- or (*S*)-1-Amino-2-propanol | 75.11 | 9.6 | 99.37 | + | -3.12 |
| (*R*)- or (*S*)-2-Amino-1-propanol | 75.11 | 9.79 | 99.6 | + | -3.3 |
| (*R*)- or (*S*)-1,2-Diaminopropane | 74.13 | 9.83 | 99.76 | ++ (34.22) | -3.56 |
| 1,3-Diamino-2-propanol | 90.12 | 9.45 | 99.75 | ++ (71.46) | -4.66 |
| 3-Methylamino-1-propanol | 89.14 | 10 | 99.75 | + | -3.46 |
| Tris(3-aminopropyl)amine | 188.31 | 10.3 | 100 | +++ (98.69) | -9.06 |
| 3,3'-Diamino-*N*-methyldipropylamin | 145.25 | 10.14 | 100 | +++ (13.96)<br>++ (85.2) | -6.06 |
| Butylamine | 73.14 | 10.21 | 99.84 | + | -2.12 |
| tert-Butylamine | 73.14 | 10.65 | 99.94 | + | -2.82 |
| Isobutylamine | 73.14 | 10.24 | 99.85 | + | -2.14 |
| 4-Amino-1-butanol | 89.14 | 9.9 | 99.69 | + | -3.31 |
| (*R*)- or (*S*)-3-Aminobutan-1-ol | 89.14 | 10.06 | 99.78 | + | -3.49 |
| D/L Valinol | 103.17 | 9.9 | 99.68 | + | -2.53 |
| (*R*)- or (*S*)-tert-Leucinol | 117.19 | 9.61 | 99.39 | + | -1.76 |
| Amylamine | 87.17 | 10.21 | 99.84 | + | -1.73 |
| tert-Amylamine | 87.17 | 10.67 | 99.95 | + | -2.36 |
| 5-Amino-1-pentanol | 103.17 | 10.21 | 99.84 | + | -3.17 |
| (*R*)- or (*S*)-2-Amino-1-pentanol | 103.17 | 9.83 | 99.63 | + | -2.47 |
| (*R*)- or (*S*)-Leucinol | 117.19 | 9.83 | 99.63 | + | -2.14 |
| (S)-(+)-Isoleucinol | 117.19 | 9.89 | 99.68 | + | -2.12 |
| Hexylamine | 101.19 | 10.21 | 99.84 | + | -1.33 |
| 6-Amino-1-hexanol | 117.19 | 10.21 | 99.84 | + | -2.78 |
| (*R*)- or (*S*)-2-Amino-1-hexanol | 117.19 | 9.83 | 99.63 | + | -2.08 |
| Heptylamine | 115.22 | 10.21 | 99.84 | + | -0.93 |
| Heptaminol | 145.246 | 10.43 | 99.91 | + | -2.44 |
| Spermidine-(butyl-$d_8$) | 153.29 | 10.61 | 99.96 | +++ (84.74)<br>++ (15.22) | -7.65 |

MW, molecular weight; $pKa_{basic}$, positive charge at pH 7.4, and LogD at pH 7.4 were calculated using MarvinSketch® of Chemaxon, Hungary. Additional percentage of positive charge in the double or triple positively charged substances refers to the positive charge of all respective nitrogens at pH 7.4.

**Table S2. HPLC and mass spectrometric detection parameters of investigated aliphatic amines**

| Aliphatic amines | RT (min) | Q1 mass (Da) | Q3 mass (Da) | DP (V) | CE (V) | CXP (V) | Organic additive (%) | Flow rate (mL/min) |
|---|---|---|---|---|---|---|---|---|
| Triethylamine | 3 | 102.11 | 72.1 (58.1) | 66 | 23 | 14 | 3% | 0.300 |
| Diethanolamine | 2.6 | 106.16 | 70 (88) | 55 | 19 | 12 | | |
| Dimethylaminoethanol | 2.7 | 90.07 | 70.10 | 41 | 23 | 12 | | |
| *N*-Methyldiethanolamine | 2.74 | 120.16 | 58.2 (102.2) | 51 | 25 | 10 | | |
| 2-(Diethylamino)ethanol | 2.7 | 118.14 | 72 (44.2) | 71 | 23 | 12 | | |
| Propylamine | 2.7 | 60.05 | 41.1 (39) | 50 | 21 | 6 | | |
| Diisopropylamine | 3.15 | 102.16 | 60.2 (43.1) | 51 | 15 | 10 | | |
| 1,3-Diamino-2-propanol | 2.4 | 91.10 | 74 (56.1) | 41 | 14 | 13 | | |
| 3-Methylamino-1-propanol | 2.6 | 90.06 | 57.2 (41.1) | 61 | 27 | 10 | | |
| Butylamine | 3.07 | 74.05 | 41.2 (57.2) | 56 | 21 | 6 | | |
| Isobutylamine | 2.95 | 74.02 | 57.1 (41.1) | 31 | 13 | 10 | | |
| 4-Amino-1-butanol | 2.7 | 90.09 | 73.10 | 46 | 13 | 14 | | |
| (*R*)- or (*S*)-3-Aminobutan-1-ol | 2.7 | 90.09 | 55.1 (43.2) | 56 | 18 | 10 | | |
| D- or L-Valinol | 3.15 | 104.19 | 69.1 (45.2) | 53 | 15 | 12 | | |
| (*R*)- or (*S*)-tert-Leucinol | 3.8 | 118.16 | 83.1 (57.1) | 53 | 17 | 14 | | |
| Amylamine | 4.2 | 88.13 | 71.2 (43) | 48 | 13 | 12 | | |
| tert-Amylamine | 3.6 | 88.15 | 71.1 (43.1) | 40 | 13 | 12 | | |
| (*R*)- or (*S*)-2-Amino-1-pentanol | 3.2 | 104.11 | 69.2 (41.1) | 43 | 17 | 12 | | |
| (*R*)- or (*S*)-Leucinol | 3.9 | 118.20 | 83.2 (55.1) | 36 | 15 | 14 | | |
| (*S*)-(+)-Isoleucinol | 3.9 | 118.13 | 83.2 (55.1) | 46 | 15 | 14 | | |
| 6-Amino-1-hexanol | 3.2 | 118.16 | 83.20 | 45 | 15 | 14 | | |
| (*R*)- or (*S*)-2-Amino-1-hexanol | 4.1 | 118.14 | 55.1 (83.1) | 36 | 23 | 10 | | |
| Heptaminol | 4.7 | 146.22 | 128.1 (69) | 56 | 13 | 8 | | |
| Spermidine-(butyl-$d_8$) | 2.3 | 154.338 | 80.2 (120) | 41 | 23 | 14 | | |
| Hexylamine[a] | 5.4 | 102.13 | 44 (85.2) | 71 | 21 | 8 | 8% | |
| Heptylamine[b] | 4.4 | 116.17 | 41.1 (43.1) | 51 | 34 | 6 | 20% | |
| **Derivatised compounds** | | | | | | | | |
| Ethanolamine-$^{13}C_2$ | 3.2 | 234.210 | 171.2 (116.1) | 46 | 25 | 10 | 8% | 0.400 |
| 3-Amino-1-propanol | 4.05 | 246.182 | 171.2 (116.2) | 66 | 23 | 10 | | |
| Tris(2-aminoethyl)amine | 3.9 | 487.33 | 317.4 (171) | 161 | 25 | 8 | | 0.300 |
| | | 657.48 | 171.2 (214.2) | 116 | 59 | 10 | | |
| Tris(3-aminopropyl)amine | 6.15 | 529.41 | 171.3 (215.3) | 96 | 37 | 14 | | |
| | | 699.44 | 171.2 (555.4) | 111 | 77 | 12 | | |
| (*R*)- or (*S*)-1-Amino-2-propanol | 3.4 | 246.211 | 171.2 (116.2) | 61 | 25 | 10 | 20% | |
| (*R*)- or (*S*)-2-Amino-1-propanol | 3.4 | 246.221 | 171.2 (116) | 61 | 23 | 10 | | |
| Isopropylamine | 3.95 | 230.284 | 171.1 (116.1) | 46 | 23 | 10 | | |
| (*R*)- or (*S*)-1,2-Diaminopropane | 3.4 | 415.308 | 145.1 (171.1) | 76 | 23 | 8 | | |
| tert-Butylamine | 6.3 | 244.243 | 171.2 | 51 | 25 | 10 | | |
| 5-Amino-1-pentanol[c] | 3.4 | 274.181 | 171.1 (116.1) | 66 | 25 | 10 | | |

| | | | | | | | |
|---|---|---|---|---|---|---|---|
| 3,3′-Diamino-*N*-methyldipropylamin | 2.3 | 486.30 | 342.4 (171.2) | 86 | 23 | 10 | |
| **Internal standards** | | | | | | | |
| Buformin | 3.9 | 158 | 60 | 40 | 35 | 10 | 3% |
| Ranitidine-$d_6$ | 3.9 | 321.2 | 176 (130.1) | 65 | 25 | 15 | 8% |
| Fenoterol-$d_6$ | 3.4 | 310.3 | 109.1 (141) | 71 | 40 | 12 | 20% |
| Isopropylamine | 3.95 | 246.221 | 171.1 (116.1) | 46 | 23 | 10 | |

Abbreviations: RT, retention time; Q1, first quadrupole; Q3, third quadrupole: quantifier (qualifier); DP, declustering potential; CE, collision energy; CXP, collision cell exit potential

[a]quantified with ranitidine-$d_6$ as an IS

[b]quantified with fenoterol-$d_6$ as an IS

[c]quantified with isopropylamine as an IS

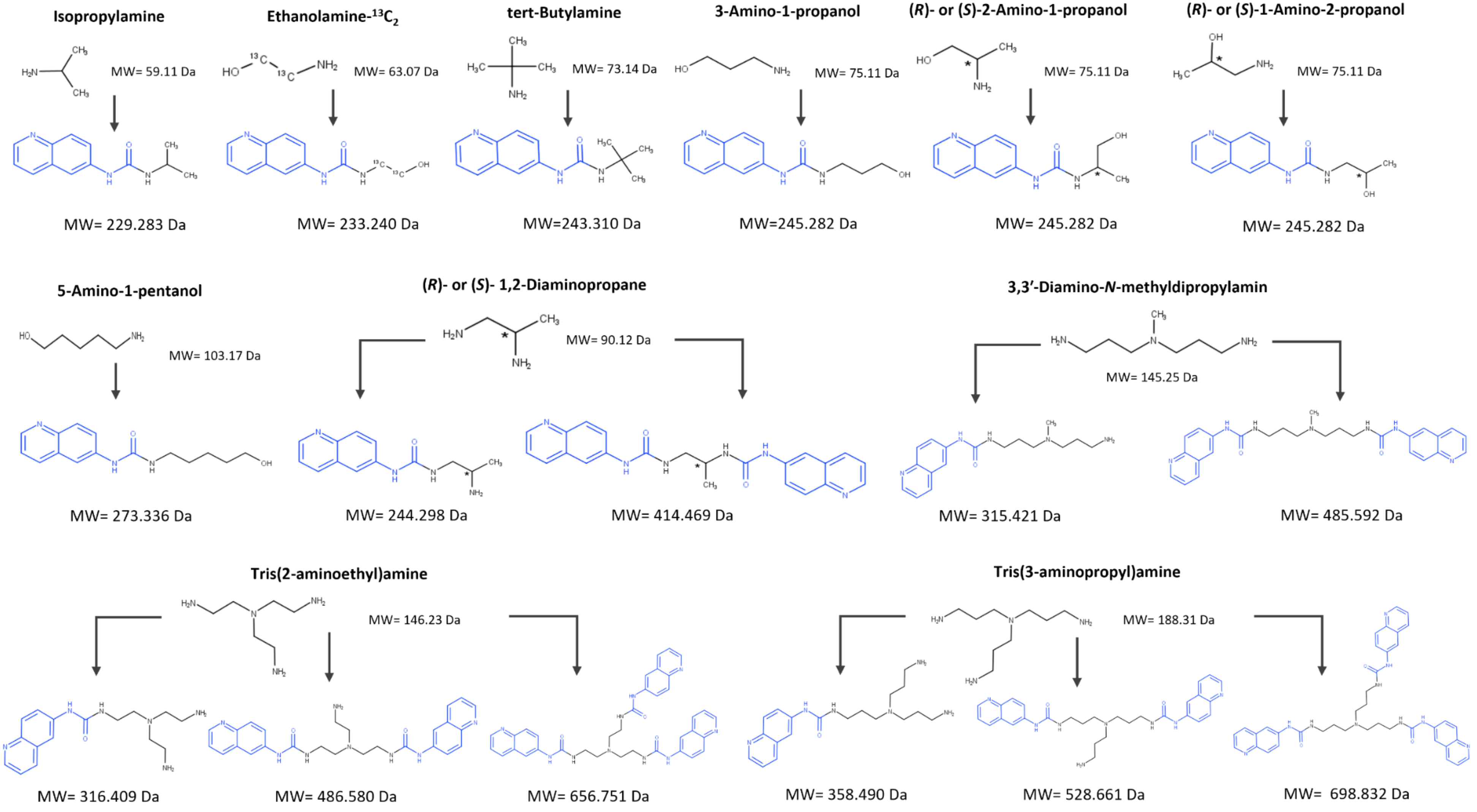
Isopropylamine
MW= 59.11 Da
MW= 229.283 Da
Ethanolamine-13C2
MW= 63.07 Da
MW= 233.240 Da
tert-Butylamine
MW= 73.14 Da
MW=243.310 Da
3-Amino-1-propanol
MW= 75.11 Da
MW=245.282 Da
(R)- or (S)-2-Amino-1-propanol
MW= 75.11 Da
MW= 245.282 Da
(R)- or (S)-1-Amino-2-propanol
MW= 75.11 Da
MW= 245.282 Da
5-Amino-1-pentanol
MW= 103.17 Da
MW= 273.336 Da
(R)- or (S)- 1,2-Diaminopropane
MW= 90.12 Da
MW= 244.298 Da
MW= 414.469 Da
3,3′-Diamino-N-methyldipropylamin
MW= 145.25 Da
MW= 315.421 Da
MW= 485.592 Da
Tris(2-aminoethyl)amine
MW= 146.23 Da
MW= 316.409 Da
MW= 486.580 Da
MW= 656.751 Da
Tris(3-aminopropyl)amine
MW= 188.31 Da
MW= 358.490 Da
MW= 528.661 Da
MW= 698.832 Da

**Figure S1.** Amines subjected to the derivatisation reaction and all of their possible derivatisation products, along with the molecular weights before and after the derivatisation reaction. Chiral centres are marked with an asterisk. Structures were depicted and the molecular weights calculated using MarvinSketch® (version 24.1.3; Chemaxon, Budapest, Hungary).
